# Serious adverse reaction associated with the COVID-19 vaccines of BNT162b2, Ad26.COV2.S, and mRNA-1273: Gaining insight through the VAERS

**DOI:** 10.3389/fphar.2022.921760

**Published:** 2022-11-07

**Authors:** Ming-Ming Yan, Hui Zhao, Zi-Ran Li, Jun-Wei Chow, Qian Zhang, Yu-Peng Qi, Shu-Shan Wu, Ming-Kang Zhong, Xiao-Yan Qiu

**Affiliations:** ^1^ Clinical Pharmacy Department, Huashan Hospital, Fudan University, Shanghai, China; ^2^ School of Pharmacy, Fudan University, Shanghai, China; ^3^ University of Nebraska Medical Center College of Pharmacy, Omaha, United States

**Keywords:** COVID-19 vaccine safety, adverse events following immunization, reporting odds ratio, BNT162b2, Ad26.Cov2.S, mRNA-1273

## Abstract

**Background and purpose:** Serious adverse events following immunization (AEFI) associated with the COVID-19 vaccines, including BNT162b2 (Pfizer-BioNTech), Ad26.COV2.S (Janssen), and mRNA-1273 (Moderna), have not yet been fully investigated. This study was designed to evaluate the serious AEFI associated with these three vaccines.

**Methods:** A disproportionality study was performed to analyze data acquired from the Vaccine Adverse Event-Reporting System (VAERS) between 1 January 2010 and 30 April 2021. The reporting odds ratio (ROR) method was used to identify the association between the COVID-19 vaccines BNT162b2, Ad26.COV2.S, and mRNA-1273 and each adverse event reported. Moreover, the ratio of the ROR value to the 95% CI span was applied to improve the credibility of the ROR. The median values of time from vaccination to onset (TTO) for the three vaccines were analyzed.

**Results:** Compared with BNT162b2 and mRNA-1273, Ad26.COV2.S vaccination was associated with a lower death frequency (*p* < 0.05). Ad26.COV2.S vaccination was associated with a lower birth defect and emergency room visit frequency than BNT162b2 (*p* < 0.05). There were 6,605, 830, and 2,292 vaccine recipients who suffered from COVID-19-related symptoms after vaccination with BNT162b2, Ad26.COV2.S, and mRNA-1273, respectively, including people who were infected by COVID-19, demonstrated a positive SARS-CoV-2 test, and were asymptomatic. Serious AEFI, including thromboembolism, hemorrhage, thrombocytopenia, cardiac arrhythmia, hypertension, and hepatotoxicity, were associated with all three vaccines. Cardiac failure and acute renal impairment events were associated with BNT162b2 and mRNA-1273, while seizure events were associated with BNT162b2 and Ad26.COV2.S. The median values of TTO associated with the three vaccinations were similar.

**Conclusion:** These findings may be useful for health workers and the general public prior to inoculation, especially for patients with underlying diseases; however, the risk/benefit profile of these vaccines remains unchanged. The exact mechanism of SARS-CoV-2 vaccine-induced AEFI remains unknown, and further studies are required to explore these phenomena.

## 1 Introduction

The global COVID-19 epidemic is still very serious. As of July 2021, more than 196,601,716 confirmed cases and 4,198,083 deaths have been registered in more than 200 countries and territories. The current global case fatality rate of COVID-19 is 2.1%, and the cure rate is 90.4%. In particular, more than 10,000 cases of COVID-19 pneumonia are reported every day in more than 10 countries (WHO, 2021b). In October 2020, a new virus variant, Delta, was discovered for the first time in India. The Delta strain is more infectious than previous strains, and infected individuals are more likely to develop serious illness (Reardon, 2021). Delta spread to 74 countries and gradually became the main epidemic strain. Recently, a new variant of COVID-19 was reported from South Africa. The World Health Organization (WHO) named this mutant Omicron (B.1.1.529) as a variant of concern on 26 November 2021(Karim and Karim, 2021).

There is currently no effective treatment for the novel coronavirus, and vaccination is still one of the most economical and effective measures to prevent infection. Since the outbreak of COVID-19, countries worldwide have encouraged the development of vaccines. As of 27 July 2021, 108 vaccines have entered clinical trials, of which 27 have entered phase 3 clinical trials and eight have entered phase 4 clinical trials (WHO, 2021a). There are three main kinds of COVID-19 vaccines on the market: inactivated vaccines, adenovirus vector vaccines, and mRNA vaccines, including Pfizer’s mRNA vaccine—BNT162b2, Moderna’s mRNA-1273, Janssen’s adenovirus vector vaccine—Ad26.COV2.S, and China Bio’s novel coronavirus-inactivated vaccine (Bok et al., 2021). The development cycle of COVID-19 vaccines is short, and adverse reactions need to be monitored.

Although the abovementioned three vaccines can reduce the infection rate and the symptoms of infected patients, they can also elicit numerous adverse reactions, according to several researchers (Andraska et al., 2021; Jeet Kaur et al., 2021; See et al., 2021). The results of existing clinical studies on the COVID-19 vaccine show that the vast majority of adverse reactions are expected to be mild. Meanwhile, the incidence of unintended adverse reactions is low, and most of them are mild to moderate and mainly local reactions. Common local adverse reactions to the aforementioned three COVID-19 vaccines include pain, pruritus, redness, and induration at the injection site. Systemic adverse reactions mainly include fatigue, fever, diarrhea, cough, muscle pain, joint pain, headache, nausea, anorexia, and chills (Chen et al., 2021). Most of these adverse reactions occur in the first 24–48 h after vaccination (Rolla et al., 2021).

However, serious or rare adverse reactions have been reported. BNT162b2 and mRNA-1273 vaccines can cause serious allergic reactions (Shimabukuro and Nair, 2021; Shimabukuro and Surgeons, 2021), usually within 30 min of vaccination; therefore, contraindications and history of allergy should be determined in advance, and adequate amounts of epinephrine should be available. Serious adverse reactions associated with the Ad26.COV2.S vaccine include serious skin adverse reactions (Lospinoso et al., 2021), Bell’s palsy (Nishizawa et al., 2021), and anxiety (Hause et al., 2021). Some rare adverse reactions have been reported with Pfizer-BioNTech and mRNA-1273 vaccines, including cutaneous adverse reactions (Larson et al., 2021), myasthenia gravis (Tagliaferri et al., 2021), hearing loss (Wichova et al., 2021), rhabdomyolysis (Mack et al., 2021), urinary tract infections (Zhao et al., 2021), liver damage (Ackerman et al., 2021), gastroparesis (Scott et al., 2021), and acute pancreatitis (Parkash et al., 2021). If these events are not properly handled, they can lead to adverse effects in individuals and serious consequences for the prevention and control of infectious diseases. Therefore, it is of great significance to explore the possible signals of adverse reactions to vaccines and predict and avoid the occurrence of adverse events.

The serious adverse events following immunization (AEFI) reported concerning the COVID-19 vaccines also include thrombosis (Marcucci et al., 2021), thrombocytopenia (Welsh et al., 2021), and several cases of unusual thrombotic events in combination with thrombocytopenia (Gunther et al., 2021; Iavorska et al., 2021). The vast majority of reported immune thrombotic thrombocytopenia (VITT) cases have occurred following adenoviral vector-based vaccination with ChAdOx1nCoV-19 (Oxford/AstraZeneca) (Bayas et al., 2021; Schultz et al., 2021) or Ad26.COV2.S (Bayas et al., 2021; Iavorska et al., 2021; Schultz et al., 2021; See et al., 2021). It is unclear whether thrombosis is related to the Pfizer-BioNTech and mRNA-1273 vaccines or whether BNT162b2 and Ad26.COV2.S recipients have a similar frequency of blood clots and bleeding. It is also unclear whether patients are at risk of other serious AEFI when receiving the COVID-19 vaccine.

The U.S. Food and Drug Administration (FDA)’s Vaccine Adverse Event Reporting System (VAERS) is the database for vaccines. The VAERS was used to obtain data, and statistical methods such as the reporting odds ratio (ROR) were used to process the collected data to discover the various adverse reaction signals of the aforementioned three COVID-19 vaccines, especially serious AEFI. We hope that the results may enhance physicians’ awareness of the potential serious side effects associated with COVID-19 vaccines.

## 2 Methods

### 2.1 Data source

On 8 May 2021, we obtained VAERS data from the U.S. FDA website (https://vaers.hhs.gov). Individuals who were vaccinated between 1 January 2010 and 30 April 2021 and were 18 years of age or older at the time of vaccination were included in the study. Each report was categorized based on the following binomial factors: “with” or “without” exposure to the administration of vaccines of interest (specifically, BNT162b2 from Pfizer-BioNTech, mRNA-1273 from Moderna, and Janssen’s adenovirus vector vaccine—Ad26.COV2.S) and “with” or “without” the experience of an AEFI category of interest.

### 2.2 Definition of target adverse events

Serious AEFI were defined as hospitalization, extended hospital stay, suffering from life-threatening events, admission to an emergency department, disability related to vaccination, birth defect, and death. The recovery from AEFI was also statistically analyzed. Other serious AEFI including thromboembolism, hemorrhage, thrombocytopenia, cardiac arrhythmias, cardiac failure, hypertension, hepatotoxicity, acute renal impairment, acute pancreatitis, and convulsion events were also analyzed. The preferred terms (PTs) of these serious AEFI were defined based on the Standardized MedDRA Query (SMQ, version 23.0) (Almenoff et al., 2005). The PTs of COVID-19-related events included “COVID-19,” “COVID-19 pneumonia,” “exposure to SARS-CoV-2,” “suspected COVID-19,” “asymptomatic COVID-19,” “COVID-19 immunization,” “SARS-CoV-2 test,” “SARS-CoV-2 test negative,” “SARS-CoV-2 test positive,” and “SARS-CoV-2 antibody test negative.” The other PTs used in this study are listed in Supplementary Tables S1–S10.

### 2.3 Disproportionality analyses and signal detection

The ROR method of disproportionality based on a four-grid algorithm was first proposed by the Netherlands Pharmacovigilance Center and has the advantages of high sensitivity, simple calculation, and the ability to eliminate a large number of biases (Napke, 1968; Moore et al., 2005). When a target adverse effect is caused by a COVID-19 vaccine, it will occur more frequently in reports listing COVID-19 vaccines than in those not listing COVID-19 vaccines, as suspect or concomitant, thus generating disproportionality (Almenoff et al., 2007). In the disproportionality analysis, a statistically significant ROR was defined as reported: absolute count of PTs linked to the targeted drug ≥ 3 and a lower limit of the 95% confidence interval (CI) > 1 (Wilson et al., 2004).

The ROR value tends to become extraordinarily large with a broad 95% CI because of a minor-valued absolute count (a), which is a property of the case/non-case method. A unique method was used to improve the credibility of the ROR by removing false positives caused by lack of absolute counts. The 95% CI reflects the potential range in which the ROR value can fluctuate since the ROR value indicates the signal’s intensity (Kadjo et al., 2017). The ratio of the ROR value to the span of 95% CI might be considered the height (S/W = H) of the imaginary peak if we regard the signal, represented as ROR value (S), as an imagined peak of normal distribution and the span of 95% CI as the base of its peak (W). A sharp peak or spike with a large H value and a minor W value could be described as a positive signal. H values greater than 0.5 were considered the threshold of a legitimate signal using the ROR exclusion criterion (absolute count a ≥ 3, the lower end of 95% CI ≥ 1). The higher the peak, the stronger the association between the suspected drug and the adverse effect of concern.

### 2.4 Data processing and statistical analysis

We took a step further to calculate the total frequencies per 1,000 reports of death, life-threatening events, birth defects, emergency department visits, hospitalizations, extended hospital stays, and disabilities related to vaccination between the three COVID-19 vaccines and all the other vaccines in order to investigate whether there was a statistical difference among the three vaccines. Serious adverse events and main complications following immunization associated with the three COVID-19 vaccines were also analyzed in order to observe the effects of serious AEFI. In order to study the occurrence of thrombosis with hemorrhage and thrombocytopenia, the number of hemorrhage and thrombocytopenia events occurring simultaneously with thrombosis was analyzed. The median value of time from the vaccination to the onset (TTO) for the COVID-19 vaccines BNT162b2, Ad26.COV2.S, and mRNA-1273 was analyzed. When the TTO was statistically described, cases with TTO ≥ 957 days were cast as error data (count from the first reported case on 27 December 2019 until now), and TTO ≥ 365 days was kept but with doubts.

In the data mining process, malignancy PTs were automatically compared and analyzed by MATLAB version R2019a (MathWorks, USA). The statistical package SPSS version 19.0 (Statistical Product and Service Solutions) was used for statistical analyses. Using the chi-square test, *p*-values < 0.05 were considered statistically significant. RORs, 95% CIs, and the frequencies of AEFI reports associated with the three COVID-19 vaccines were calculated using SPSS.

## 3 Results

### 3.1 Descriptive analyses of serious AEFI related to BNT162b2, Ad26.COV2.S, and mRNA-1273

**FIGURE 1:**
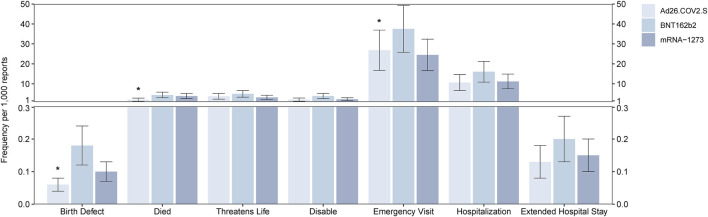
Report frequency of deaths, life-threatening events, birth defects, disabilities, emergency room visit, hospitalization events, and extended hospital stay events after vaccination of the BNT162b2, Ad26.COV2.S, and mRNA-1273. *means *p* < 0.05 after the chi-square test.

A total of 445,926, 167,457, and 535,126 cases of AEFI were reported after vaccination with BNT162b2, Ad26.COV2.S, and mRNA-1273, respectively. Among the serious AEFI associated with the COVID-19 vaccine, people who died after the vaccination accounted for 1,963 cases (4.40 per 1,000, 1,963/445,926) for BNT162b2, 345 cases (2.06 per 1,000, 345/167,457) for Ad26.COV2.S, and 2,077 cases (3.88 per 1,000, 2,077/535,126) for mRNA-1273. The frequency of deaths was statistically different between Ad26.COV2.S and the other two vaccines (*p* < 0.05). Regarding birth defects associated with the COVID-19 vaccines, 79 cases (0.18 per 1,000) for BNT162b2, 10 cases (0.06 per 1,000) for Ad26.COV2.S, and 53 cases (0.10 per 1,000) for mRNA-1273 were reported. The reported frequency of birth defects was statistically different between Ad26.COV2.S and BNT162b2 (*p* < 0.05). A total of 16,737 patients who went to the emergency room (37.53 per 1,000) for BNT162b2, 4,494 (26.84 per 1,000) for Ad26.COV2.S, and 13,092 (24.46 per 1,000) for mRNA-1273 were reported. The reported frequency of emergency room visits was statistically different between Ad26.COV2.S and BNT162b2 (*p* < 0.05). For the reported frequency of life-threatening AEFI, hospitalization, extended hospital stays, and disability related to vaccination, no statistical difference was observed among the three vaccines, which is shown in Figure 1.

Among the 1,963 people who died after receiving the BNT162b2 vaccine, the most common combined AEFI were dyspnea (accounting for 12.48% of the 1,963 people who died after the vaccination), cardiac arrest (8.51%), and lack of response to stimuli (7.74%). One hundred and forty-eight patients died of COVID-19 (7.54%), and 140 patients showed a positive SARS-CoV-2 test (7.13%). 2221 people (4.98 per 1,000, 2,221/445,926) suffered from life-threatening AEFI, with dyspnea (19.18%), pulmonary embolism (11.80%), and headache (9.32%) reported with high frequency. The numbers of patients who went to the emergency room and were hospitalized after vaccination were 16,737 (37.53 per 1,000, 16,737/445,926) and 7,151 (16.04 per 1,000, 7,151/445,926), respectively, because of dizziness (18.30%), dyspnea (15.57%, with 15.84% for hospital visits), headache (13.16%), and nausea (12.03%). The serious AEFI associated with the three vaccines and the main complications are presented in Supplementary Tables S11–S13.

The median values of TTO associated with the three vaccinations were similar, and this is illustrated in Supplementary Table S14.

### 3.2 COVID-related events

**FIGURE 2:**
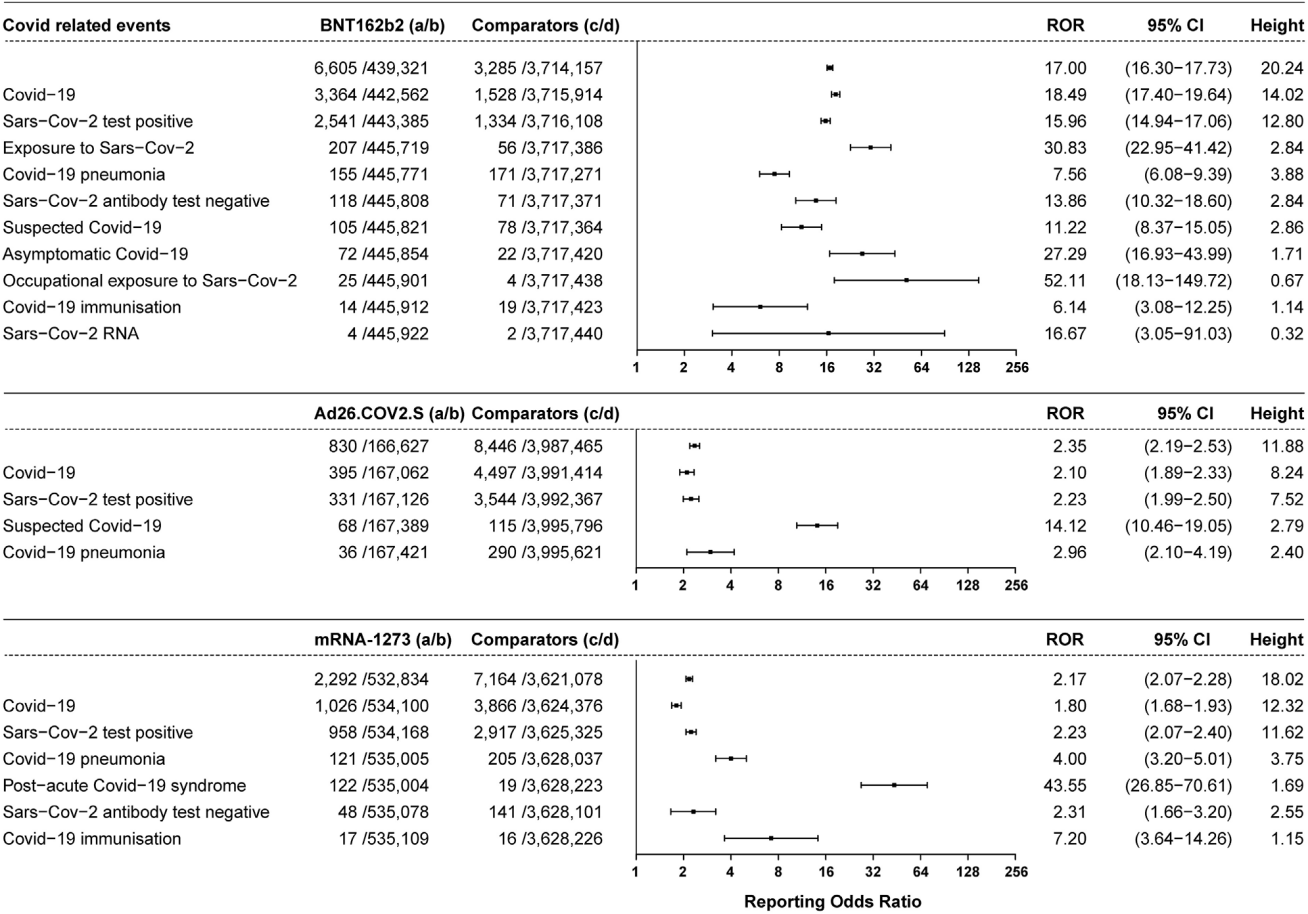
ROR values and height of the COVID-19 vaccines of BNT162b2-, Ad26.COV2.S-, and mRNA-1273-associated COVID events. a: cases of target COVID events reported concerning the target COVID-19 vaccines; b: cases of other AEFI reported concerning the target COVID-19 vaccines; c: cases of target COVID events reported concerning all the other vaccines; d: cases of other AEFI reported concerning all the other vaccines.

A total of 6605 vaccine recipients suffered from COVID-19-related symptoms (ROR 17.00, 95% CI 16.30–17.73, height 20.24), accounting for 1.48% of all the AEFI reported for BNT162b2 (6,605/445,926). A total of 3,364 cases of people suffering from COVID-19 symptoms were reported (ROR 18.49, 95% CI 17.40–19.64, height 14.02). A total of 155 patients were diagnosed with COVID-19 pneumonia (ROR 7.56, 95% CI 6.08–9.39, height 3.88), 105 patients were suspected COVID-19 (ROR 11.22, 95% CI 8.37–15.05, height 2.86), and there were 72 cases of asymptomatic COVID-19 (ROR 27.29, 95% CI 16.93–43.99, height 1.71) after vaccination with BNT162b2. A total of 2,541 cases of a positive SARS-CoV-2 test were reported (ROR 15.96, 95% CI 14.94–17.06, height 12.80), accounting for 0.57% of all the AEFI reported for BNT162b2 (2,541/445,926), and 118 cases showed a negative SARS-CoV-2 antibody test (ROR 13.86, 95% CI 10.32–18.60, height 2.84) after vaccination with BNT162b2. A total of 830 vaccine recipients suffered from COVID-19-related symptoms (ROR 2.35, 95% CI 2.19–2.53, height 11.88) after vaccination with Ad26.COV2.S. A total of 395 reported cases of COVID-19 (ROR 2.10, 95% CI 1.89–2.33, height 8.24), 331 cases of positive SARS-CoV-2 tests (ROR 2.23, 95% CI 1.99–2.50, height 7.52), 36 cases of COVID-19 pneumonia (ROR 2.96, 95% CI 2.10–4.19, height 2.40), and 68 cases of suspected COVID-19 (ROR 14.12, 95% CI 10.46–19.05, height 2.79) after vaccination with Ad26.COV2.S were reported. In total, 2,292 COVID-19-related symptoms were reported related to mRNA-1273 (ROR 2.17, 95% CI 2.07–2.28, height 18.02). A total of 1026 cases of COVID-19 (ROR 1.80, 95% CI 1.68–1.93, height 12.32), 958 cases of positive SARS-CoV-2 tests (ROR 2.23, 95% CI 2.07–2.40, height 11.62), 121 cases of COVID-19 pneumonia (ROR 4.00, 95% CI 3.20–5.01, height 3.75), 122 cases of post-acute COVID-19 syndrome (ROR 43.55, 95% CI 26.85–70.61, height 1.69), and 48 cases of negative SARS-CoV-2 antibody tests were reported (ROR 2.31, 95% CI 1.66–3.20, height 2.55) after vaccination with mRNA-1273. The COVID-related events associated with the three vaccines are shown in Figure 2.

### 3.3 Coagulation disorder-related events

#### 3.3.1 Thrombotic events

**FIGURE 3:**
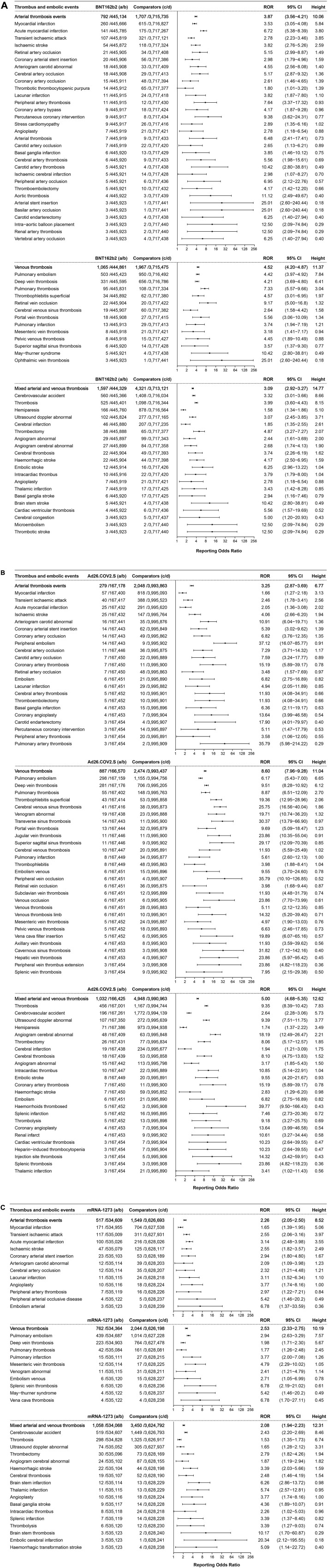
**(A)** ROR values and height of the COVID-19 vaccines of BNT162b2-associated thrombotic events. a: cases of target thrombotic events reported concerning the BNT162b2 vaccine; b: cases of other adverse events reported concerning the BNT162b2 vaccine; c: cases of target thrombotic events reported concerning all the other vaccines; d: cases of adverse events reported concerning all the other vaccines.

Thrombotic events were shown to be associated with BNT162b2, Ad26.COV2.S, and mRNA-1273 vaccines (Figures 3A–C). A total of 3,454 cases of thrombotic events were associated with the BNT162b2 vaccine (with a frequency of 7.75 per 1,000, 3,454/445,926), including 792 cases of arterial thrombotic events with an ROR of 3.87 (95% CI 3.56–4.21, height 10.06), ranked by the absolute number of cases including myocardial infarction (ROR 3.53, 95% CI 3.05–4.08, height 5.84, 260 cases), acute myocardial infarction (ROR 6.72, 95% CI 5.38–8.39, height 3.80, 141 cases), and transient ischemic attack (ROR 2.78, 95% CI 2.23–3.46, height 3.85, 107 cases). In total, 1,065 cases of venous thrombosis were associated with BNT162b2 vaccines with an ROR of 4.52 (95% CI 4.20–4.87, height 11.37), including pulmonary embolism (ROR 4.42, 95% CI 3.97–4.92, height 7.84, 503 cases), deep vein thrombosis (ROR 4.21, 95% CI 3.69–4.80, height 6.41, 331 cases), and pulmonary thrombosis (ROR 7.33, 95% CI 5.57–9.66, height 3.04, 95 cases). A total of 1,597 cases of mixed arterial and venous thrombosis were reported for BNT162b2 vaccines with an ROR of 3.09 (95% CI 2.92–3.27, height 14.77), with the top three absolute number of cases including cerebrovascular accident (ROR 3.32, 95% CI 3.01–3.66, height 8.66, 560 cases), thrombosis (ROR 3.99, 95% CI 3.60–4.43, height 8.15, 525 cases), and hemiparesis (ROR 1.58, 95% CI 1.34–1.86, height 5.10, 166 cases).

A total of 2198 cases of thrombotic events were associated with the Ad26.COV2.S vaccine, with a frequency of 13.13 per 1,000 (2,198/167,457). A total of 279 cases of arterial thrombosis events with an ROR value of 3.25 (95% CI 2.87–3.69, height 6.77) including myocardial infarction (ROR 1.66, 95% CI 1.27–2.18, height 3.13, and 57 cases) and transient ischemic attack (ROR 2.46, 95% CI 1.78–3.41, height 2.56) were reported at the top. A total of 887 cases of venous thrombosis with an ROR of 8.60 (95% CI 7.96–9.28, height 11.04), including pulmonary embolism with an ROR value of 6.17 (95% CI 5.43–7.00, height 6.65, 298 cases) and deep vein thrombosis with an ROR of 9.51 (95% CI 8.28–10.92, height 6.12, 281 cases) were ranked in the top. For mixed arterial and venous thrombosis, 1,032 cases were reported (ROR 5.00, 95% CI 4.68–5.35, height 12.62), including thrombosis (ROR 9.35, 95% CI 8.39–10.42, height 7.83, 456 cases), cerebrovascular accident (ROR 2.64, 95% CI 2.28–3.06, height 5.73, 196 cases), and abnormal ultrasound (ROR 9.39, 95% CI 7.51–11.75, height 3.77, 107 cases).

A total of 2,337 cases of thrombotic events were associated with mRNA-1273, with a frequency of 4.37 per 1,000 (2,337/535,126). A total of 517 cases of arterial thrombosis events with an ROR value of 2.26 (95% CI 2.05–2.50, height 8.52), including myocardial infarction (ROR 1.65, 95% CI 1.39–1.95, height 5.06, 171 cases), transient ischemic attack (ROR 2.55, 95% CI 2.06–3.16, height 3.97, 117 cases), and acute myocardial infarction (ROR 3.14, 95% CI 2.48–3.98, height 3.55, 100 cases), were reported. A total of 762 cases of venous thrombosis, with an ROR of 2.53 (95% CI 2.33–2.75, height 10.19) and pulmonary embolism with an ROR value of 2.94 (95% CI 2.63–3.29, height 7.57, 439 cases) and deep vein thrombosis with an ROR of 1.98 (95% CI 1.71–2.30, height 5.67, 223 cases) were ranked in the top. A total of 1,058 cases of mixed arterial and venous thrombosis reported (ROR 2.08, 95% CI 1.94–2.23, height 12.31); cerebrovascular accident (ROR 2.43, 95% CI 2.20–2.69, height 8.46, 519 cases) and thrombosis (ROR 1.53, 95% CI 1.35–1.73, height 6.74, 298 cases) were ranked in the top.

#### 3.3.2 Hemorrhage events

**FIGURE 4:**
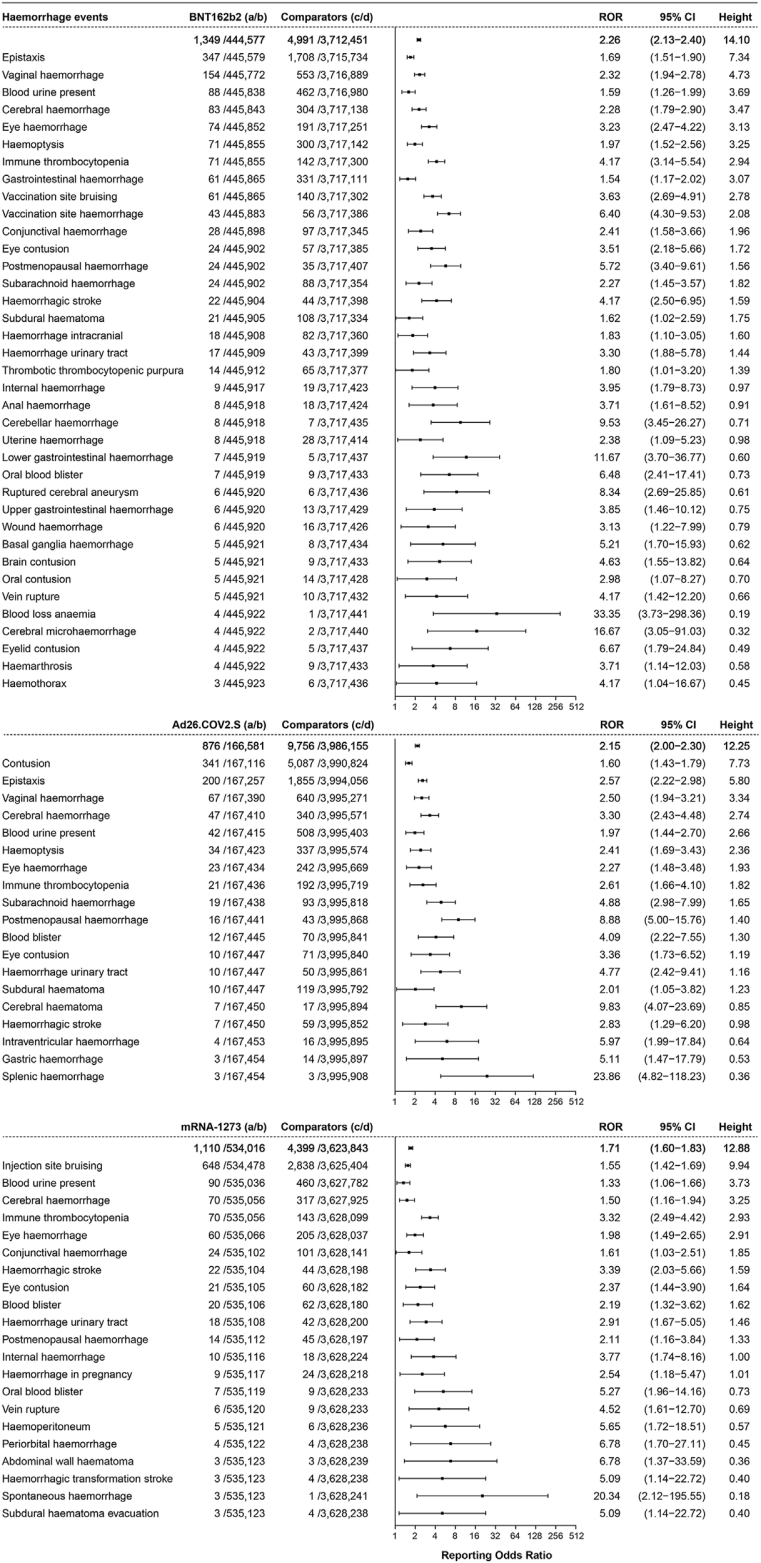
ROR values and height of the COVID-19 vaccines of BNT162b2-, Ad26.COV2.S-, and mRNA-1273-associated hemorrhage events. a: cases of target hemorrhage events reported concerning the target COVID-19 vaccines; b: cases of other adverse events reported concerning the target COVID-19 vaccines; c: cases of target hemorrhage events reported concerning all the other vaccines; d: cases of other adverse events reported concerning all the other vaccines.

Hemorrhage events were reported and showed association with the three COVID-19 vaccines, as shown in Figure 4. A total of 1,349 cases of hemorrhage events were associated with BNT162b2, with an ROR of 2.26 (95% CI 2.13–2.40, height 14.10), and epistaxis (ROR 1.69, 95% CI 1.51–1.90, height 7.34, 347 cases) and vaginal hemorrhage (ROR 2.32, 95% CI 1.94–2.78, height 4.73, 154 cases) were ranked in the top two. A total of 876 cases of hemorrhage events were reported to be associated with Ad26.COV2.S, with an ROR of 2.15 (95% CI 2.00–2.30, height 12.25), and contusion (ROR 1.60, 95% CI 1.43–1.79, height 7.73, 341 cases) and epistaxis (ROR 2.57, 95% CI 2.22–2.98, height 5.80, 200 cases) were reported with high frequency. In total, 1,110 hemorrhage events were associated with mRNA-1273 (ROR 1.71, 95% CI 1.60–1.83, height 12.88), and injection site bruising (ROR 1.55, 95% CI 1.42–1.69, height 9.94, 648 cases) was the most frequent event.

#### 3.3.3 Thrombocytopenia events

**FIGURE 5:**
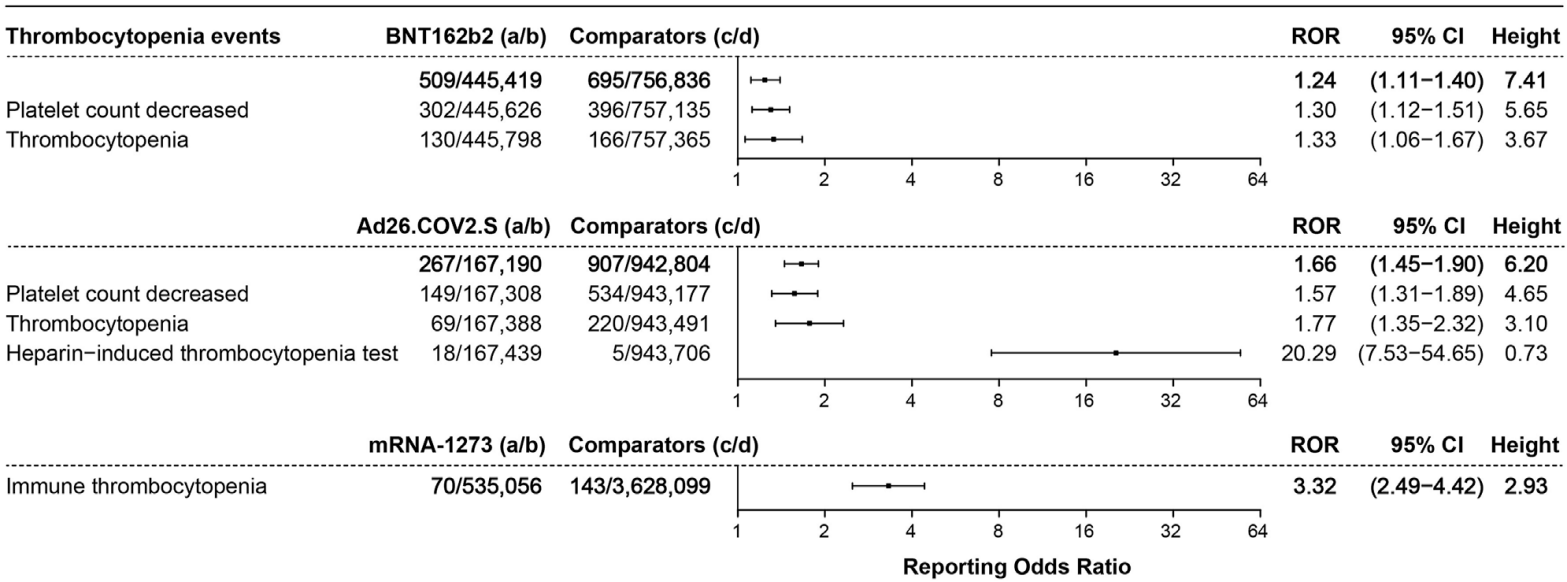
ROR values and height of the COVID-19 vaccines of BNT162b2-, Ad26.COV2.S-, and mRNA-1273-associated thrombocytopenia events. a: cases of target thrombocytopenia events reported concerning the target COVID-19 vaccines; b: cases of other adverse events reported concerning the target COVID-19 vaccines; c: cases of target thrombocytopenia events reported concerning all the other vaccines; d: cases of other adverse events reported concerning all the other vaccines.

In total, 509 cases of thrombocytopenia events were associated with BNT162b2, with an ROR of 1.24 (95% CI 1.11–1.40, height 7.41), 267 cases were associated with Ad26.COV2.S (ROR 1.66, 95% CI 1.45–1.90, height 6.20), and 70 cases of immune thrombocytopenia events were associated with mRNA-1273 (ROR 3.32, 95% CI 2.49–4.42, height 2.93), as shown in Figure 5.

#### 3.3.4 Concurrent thrombosis with hemorrhage or thrombocytopenia

A total of 329 cases of hemorrhage events occurred during the course of thrombosis, including 81, 77, 42, 33, 22, 18, and 15 cases of hemorrhage events concurrent with pulmonary embolism, thrombosis, cerebrovascular accident, deep vein thrombosis, hemorrhagic stroke, acute myocardial infarction, and hemiparesis, respectively, after administration of the BNT162b2 COVID-19 vaccine. A total of seven, six, and six cases of thrombocytopenia events occurring simultaneously with acute myocardial infarction, thrombosis, and pulmonary embolism, respectively, following BNT162b2 vaccination were reported.

In total, 235 cases of hemorrhage events that occurred with thrombosis, including 66, 60, 32, 21, and 18 cases of thrombosis, pulmonary embolism, deep vein thrombosis, abnormal cerebral angiogram, and cerebral venous sinus thrombosis, respectively, were reported following vaccination with Ad26.COV2.S. A total of 27, 15, 13, and 12 cases of thrombocytopenia events that occurred simultaneously with pulmonary embolism, cerebral venous sinus thrombosis, deep vein thrombosis, and thrombosis, respectively, were reported following vaccination with Ad26.COV2.S.

For mRNA-1273, 262 cases of hemorrhage events occurred with thrombosis, including 74, 44, 28, 26, and 22 cases that occurred during pulmonary embolism, thrombosis, deep vein thrombosis, cerebral venous sinus thrombosis, and hemorrhagic stroke, respectively. A total of 15, seven, six, and four cases of thrombocytopenia events that occurred simultaneously with pulmonary embolism, thrombosis, deep vein thrombosis, and acute myocardial infarction, respectively, were reported. The thrombotic events with hemorrhage and thrombocytopenia after the BNT162b2, Ad26.COV2.S, and mRNA-1273 vaccination are shown in Supplementary Figures S1A–C.

### 3.4 Cardiac disorders

#### 3.4.1 Cardiac arrhythmia events

**FIGURE 6:**
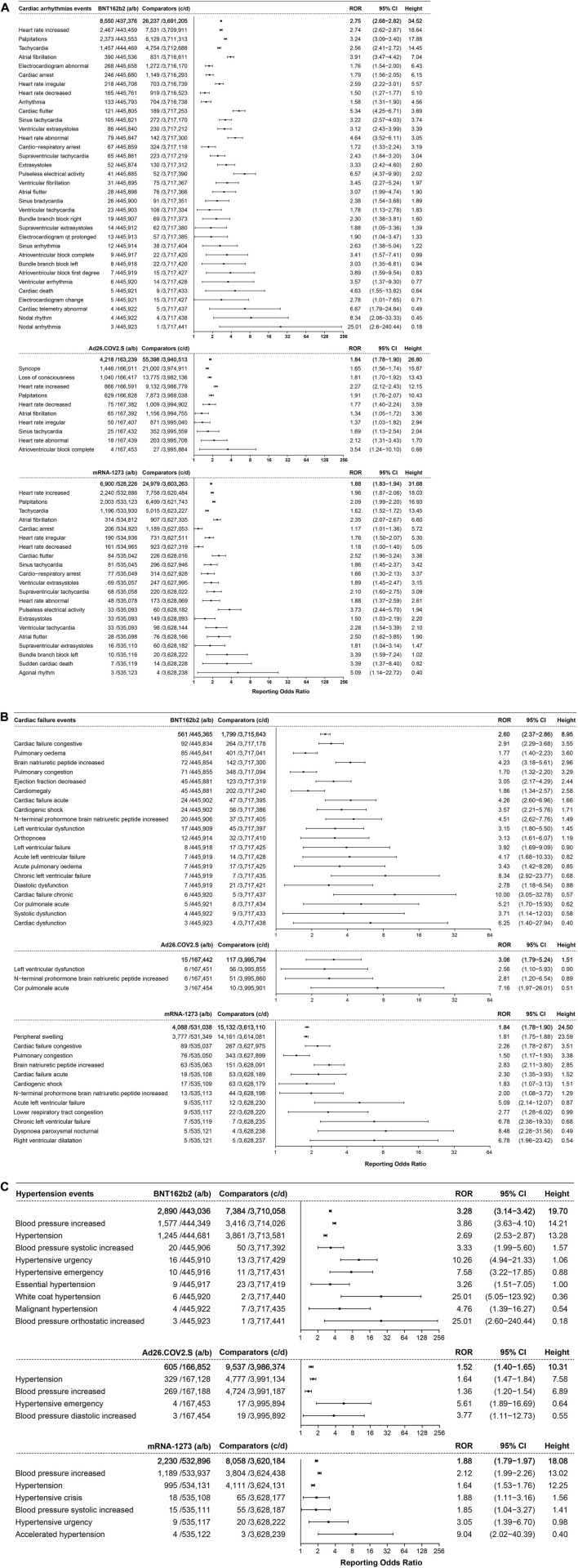
**(A)** ROR values and height of the COVID-19 vaccines of BNT162b2-, Ad26.COV2.S-, and mRNA-1273-associated cardiac arrhythmia events. a: cases of target cardiac arrhythmia events reported concerning the target COVID-19 vaccines; b: cases of other adverse events reported concerning the target COVID-19 vaccines; c: cases of target cardiac arrhythmia events reported concerning all the other vaccines; d: cases of other adverse events reported concerning all the other vaccines.

Cardiac arrhythmia events showed association with the three COVID-19 vaccines, illustrated in Figures 6A. A total of 8,550 cases of cardiac arrhythmia events were associated with BNT162b2 (ROR 2.75, 95% CI 2.68–2.82, height 34.52). Heart rate increase (ROR 2.74, 95% CI 2.62–2.87, height 18.64, 2,467 cases), palpitations (ROR 3.24 95% CI 3.09–3.40, height 17.88, 2,373 cases), and tachycardia (ROR 2.56, 95% CI 2.41–2.72, height 14.45, 1,457 cases) were ranked in the top three, with more than 1,000 cases reported. These were followed by atrial fibrillation (ROR 3.91, 95% CI 3.47–4.42, height 7.04, 390 cases), abnormal electrocardiogram (ROR 1.76, 95% CI 1.54–2.00, height 6.43, 268 cases), cardiac arrest (ROR 1.79, 95% CI 1.56–2.05, height 6.15, 246 cases), and irregular heart rate (ROR 2.59, 95% CI 2.22–3.01, height 5.57, 218 cases).

A total of 4218 cases of cardiac arrhythmia events were associated with the Ad26.COV2.S vaccine, with an ROR of 1.84 (95% CI 1.78–1.90, height 26.80), ranked by the absolute number of cases including syncope (ROR 1.65, 95% CI 1.56–1.74, height 15.87, 1,446 cases), loss of consciousness (ROR 1.81, 95% CI 1.70–1.92, height 13.43, 1,040 cases), and increased heart rate (ROR 2.27, 95% CI 2.12–2.43, height 12.15, 866 cases).

A total of 6,900 cases of cardiac arrhythmia events were associated with the mRNA-1273 vaccine, with an ROR of 1.88 (95% CI 1.83–1.94, height 31.68), ranked by absolute number of cases of increased heart rate (ROR 1.96, 95% CI 1.87–2.06, height 18.03, 2,240 cases), palpitations (ROR 2.09 95% CI 1.99–2.20, height 16.93, 2,003 cases), and tachycardia (ROR 1.62, 95% CI 1.52–1.72, height 13.45, 1,196 cases).

#### 3.4.2 Cardiac failure events

Cardiac failure events mainly showed association with BNT162b2 and mRNA-1273, as shown in Figure 6B. A total of 561 cases of cardiac failure events were associated with BNT162b2 (ROR 2.60, 95% CI 2.37–2.86, height 8.95), ranked by the absolute number of cases including congestive cardiac failure (ROR 2.91, 95% CI 2.29–3.68, height 3.55, 92 cases), pulmonary edema (ROR 1.77, 95% CI 1.40–2.23, height 3.60, 85 cases), and increased brain natriuretic peptide (ROR 4.23, 95% CI 3.18–5.61, height 2.96, 72 cases). A total of 4088 cases of cardiac failure events (ROR 1.84, 95% CI 1.78–1.90, height 24.50) showed association with mRNA-1273, of which peripheral swelling (ROR 1.81, 95% CI 1.75–1.88, height 23.59) was reported with the highest frequency. There were only 15 cases of cardiac failure events associated with Ad26.COV2.S (ROR 3.06, 95% CI 1.79–5.24, height 1.51).

#### 3.4.3 Hypertension events

The hypertension events that showed association with the three COVID-19 vaccines are illustrated in Figure 6C. A total of 2,890 cases of hypertension events were associated with BNT162b2, with an ROR of 3.28 (95% CI 3.14–3.42, height 19.70), ranked by the absolute number of cases including increased blood pressure (ROR 3.86, 95% CI 3.63–4.10, height 14.21, 1,577 cases) and hypertension (ROR 2.69, 95% CI 2.53–2.87, height 13.28, 1,245 cases). A total of 2230 cases of hypertension events were associated with mRNA-1273, with an ROR of 1.88 (95% CI 1.79–1.97, height 18.08), and increased blood pressure (ROR 2.12, 95% CI 1.99–2.26, height 13.02, 1,189 cases) was the most reported. Only 605 cases of hypertension events were associated with Ad26.COV2.S, with an ROR of 1.52 (95% CI 1.40–1.65, height 10.31).

### 3.5 Hepatotoxicity events

**FIGURE 7:**
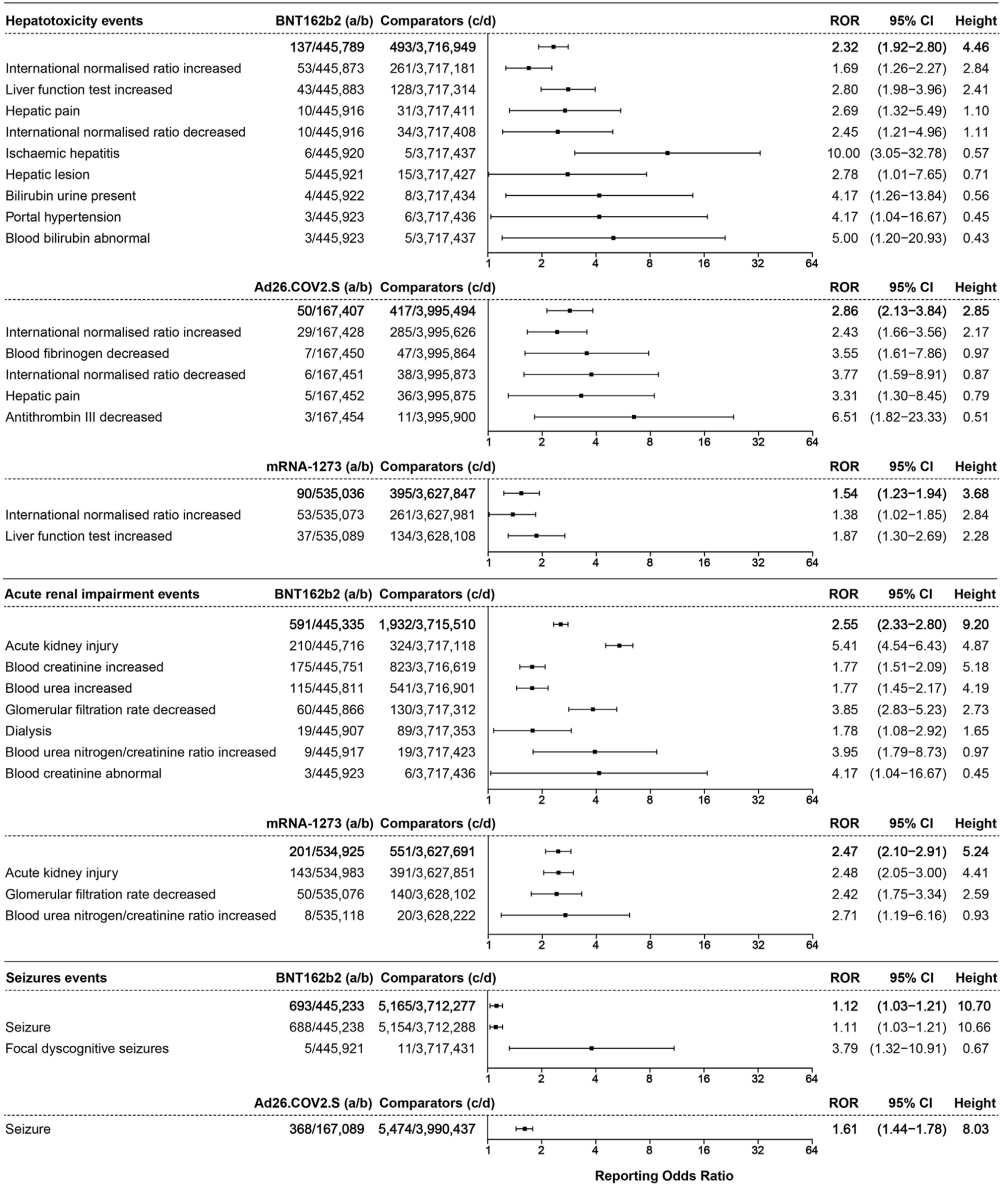
The ROR values and height of the COVID-19 vaccines of BNT162b2-, Ad26.COV2.S-, and mRNA-1273-associated hepatotoxicity, acute renal impairment, and seizure events. a: cases of target hepatotoxicity, acute renal impairment, and seizure events reported concerning the target COVID-19 vaccines; b: cases of other adverse events reported concerning the target COVID-19 vaccines; c: cases of target hepatotoxicity, acute renal impairment, and seizure events reported concerning all the other vaccines; d: cases of other adverse events reported concerning all the other vaccines.

A total of 137 cases of hepatotoxicity events were associated with BNT162b2 (ROR 2.32, 95% CI 1.92–2.80, height 4.46), with 50 cases associated with Ad26.COV2.S (ROR 2.86, 95% CI 2.13–3.84, height 2.85) and 90 cases associated with mRNA-1273 (ROR 1.54, 95% CI 1.23–1.94, height 3.68), as shown in Figure 7.

### 3.6 Acute renal impairment events

Acute renal impairment events were only associated with BNT162b2 and mRNA-1273. A total of 591 cases of acute renal impairment events were associated with BNT162b2 (ROR 2.55, 95% CI 2.33–2.80, height 9.20), ranked by the absolute number of cases including acute kidney injury (AKI) (ROR 5.41, 95% CI 4.54–6.43, height 4.87, 210 cases), increased blood creatinine (ROR 1.77, 95% CI 1.51–2.09, height 5.18, 175 cases), and increased blood urea (ROR 1.77, 95% CI 1.45–2.17, height 4.19, 115 cases). The acute renal impairment events associated with mRNA-1273 with an ROR of 2.47 (95% CI 2.10–2.91, height 5.24, 201 cases) are illustrated in Figure 7.

### 3.7 Seizure events

Seizure events only showed association with BNT162b2 and Ad26.COV2.S, as illustrated in Figure 7. A total of 693 cases of seizure events were associated with BNT162b2, with an ROR of 1.12 (95% CI 1.03–1.21, height 10.70), including seizures (ROR 1.11, 95% CI 1.03–1.21, height 10.66, 688 cases). A total of 368 cases of seizure (ROR 1.61, 95% CI 1.44–1.78, height 8.03) were associated with Ad26.COV2.S.

### 3.8 Acute pancreatitis events

A total of 57, 59, and 10 cases of acute pancreatitis events were associated with BNT162b2, mRNA-1273, and Ad26.COV2.S, respectively, but the lower limit of the 95% CI was less than 1. The results are shown in Supplementary Figure S2.

All serious AEFI associated with the BNT162b2, Ad26.COV2.S, and mRNA-1273 vaccines are summarized in Table 1.

**TABLE 1:** Severe AEFI associated with the BNT162b2, Ad26.COV2.S, and mRNA-1273 vaccines.

Severe AEFI	BNT162b2	Ad26.COV2.S	mRNA-1273
Covid events	Yes	Yes	Yes
Thrombotic events	Yes	Yes	Yes
Hemorrhage events	Yes	Yes	Yes
Thrombocytopenia events	Yes	Yes	Yes
Cardiac arrhythmia events	Yes	Yes	Yes
Cardiac failure events	Yes	Yes^a^	Yes
Hypertension events	Yes	Yes	Yes
Hepatotoxicity events	Yes	Yes	Yes
Acute renal impairment events	Yes	—	Yes
Seizure events	Yes	Yes	—
Pancreatitis events	—	—	—

^a^ Only 15 cases of cardiac failure events were associated with the Ad26.COV2.S vaccines.

## 4 Discussion

In the present study, a disproportionality analysis of VAERS data was performed, and the results demonstrated that the COVID-19 vaccines BNT162b2, mRNA-1273, and Ad26.COV2.S were associated with a broad scope of adverse events. There were statistically significant differences between Ad26.COV2.S and the other two vaccines for the frequency of deaths after vaccination. The reported frequency of birth defects and emergency room visits was statistically different between Ad26.COV2.S and BNT162b2. There were people who died after taking the three vaccines, with the most common combination of AEFI being dyspnea, cardiac arrest, and lack of response to stimuli. Patients went to the emergency rooms and visited hospitals for dizziness, dyspnea, headache, and nausea. Dyspnea, pulmonary embolism, and headache were also reported as life-threatening AEFI associated with the three vaccines. There were a few people who still developed COVID-19, showed a positive SARS-CoV-2 test, and had asymptomatic COVID-19 after vaccination. The results were in line with those of previous studies.

### 4.1 Coagulation disorders, cardiac disorders, hepatotoxicity, acute renal impairment, and seizure events associated with the three COVID-19 vaccines

The disproportionality analysis showed that BNT162b2 was associated with thrombosis, hemorrhage, thrombocytopenia, cardio arrhythmia, cardiac failure, hypertension, hepatotoxicity, acute renal impairment, and seizure events. Ad26.COV2.S showed association with thrombosis, hemorrhage, thrombocytopenia, cardiac arrhythmia, hypertension, hepatotoxicity, and seizure events. The mRNA-1273 vaccine was associated with thrombotic events, hemorrhage, thrombocytopenia, cardiac arrhythmias, cardiac failure, hypertension, hepatotoxicity, and acute renal impairment. Acute pancreatitis events showed no associations with the three vaccines because the lower limits of the 95% CIs were less than 1.

There were cases of AEFI showing association with the three vaccines, but only with a relatively large ROR value with a wide 95% CI, indicating a promising signal; however, only few cases, such as thrombotic events of arterial stent insertion, basilar artery occlusion, carotid endarterectomy, intra-aortic balloon placement, renal artery thrombosis, and vertebral artery occlusion, were associated with BNT162b2. Those signals require more reported cases to further validate them. To improve the credibility of ROR by removing false positives caused by a lack of absolute counts, we presented a unique method to verify the validity of ROR values. Those adverse events with smaller numbers of cases and wider 95% CIs usually showed a lower height.

### 4.2 Coagulation disorders following vaccination with BNT162b2, Ad26.COV2.S, and mRNA-1273 and the unknown mechanisms

The vast majority of reported cases of thrombosis are related to vaccination with ChAdOx1nCoV-19 (Bayas et al., 2021; Schultz et al., 2021) or Ad26.COV2.S (Iavorska et al., 2021; See et al., 2021). There was also a case report of cerebral venous sinus thrombosis after the Pfizer-BioNTech vaccination (Cheng, 2021). A study based on European data showed that ChAdOx1nCoV-19 and Ad26.COV2.S recipients had higher frequencies of not only serious AEFI caused by venous blood clots and hemorrhage but also thromboembolic disease and arterial events, including myocardial infarction and stroke, than those of BNT162b2 recipients (Cari et al., 2021). However, in the present study, the total thrombosis frequency of mRNA-1273 recipients was less than that of BNT162b2 and Ad26.COV2.S recipients.

To date, several studies on the mechanism of VITT caused by the COVID-19 vaccines have been reported. Published studies have suggested that the two most likely mechanisms include the effect of anti-platelet factor 4 (PF4) antibodies and the direct interaction between the adenovirus vector and platelets (Rzymski et al., 2021). Among these two mechanisms, some authors believed that the effect of PF4 antibodies was responsible for formation of the immunogenicity complex due to the negative charge on the surface of the adenovirus and the positive charge PF4 released by platelets, which further bonded with the IgG antibody, thus activating platelets and causing the additional release of PF4. PF4 interacted with the Fc-gamma receptor IIA (FcγRIIa) of platelets and then activated the platelets to enter a hypercoagulable state, leading to increased PF4 release and promoting arterial and venous thrombosis, which ultimately triggers VITT (Arepally, 2017). Another speculation was that free adenovirus DNA or some components of adenovirus could trigger VITT, but the evidence was not sufficient, and further research is needed [37]. The direct interaction between the adenovirus vector and platelets mechanism suggests that the adenoviruses in the adenovirus vector-type COVID-19 vaccine, such as human adenovirus type 5 and human adenovirus type 3, could bind to the platelets in the blood circulation through CAR and CD46 and activate platelets (Stone et al., 2007; Jin et al., 2014). Eventually, this leads to platelet aggregation, and activated platelets release a large amount of PF4, which in turn triggers VITT (Arepally et al., 2007). In addition, Rzymski et al. proposed a novel explanation referring to anti-SARS-CoV-2 spike protein antibody and PF4 cross-reaction. The author suggested that the cross-reaction of the anti-adenovirus antibody and PF4 could lead to interaction between the spike protein and platelets, causing platelets to express spike protein and activate a corresponding immune response (Rzymski et al., 2021). The proposed mechanisms of vaccine-induced immune thrombotic thrombocytopenia are shown in Supplementary Figure S3.

Notably, most of the venous thrombotic serious AEFI associated with BNT162b2 and Ad26.COV2.S vaccines were not complicated with thrombocytopenia, according to our results, suggesting that VITT is not the only type of thrombosis following the adenovirus vector. The interaction between the SARS-CoV2 virus and the angiotensin-converting enzyme 2 (ACE2) receptor as a potential cause of platelet aggregation is supported by strong evidence (Divani et al., 2020). However, the exact mechanism by which the COVID-19 vaccine might exert a prothrombotic effect without causing thrombocytopenia is still largely unknown. The binding of the spike (S) glycoprotein to ACE2 might result in ACE2 downregulation, which in turn results in higher production of angiotensin II (AngII) by ACE and less conversion of ACE2 to Ang-(1–7), which is consistent with the mechanism by which viral vector vaccines induce immunization. Stroke and thrombosis might be caused by an overactive ACE/AngII/AT1R pathway because of its prothrombotic, pro-inflammatory, and vasoconstrictor effects, especially in venous circulation (Divani et al., 2020).

### 4.3 Potential AEFI related to BNT162b2, Ad26.COV2.S, and mRNA-1273 vaccines and the unknown mechanisms

After receiving the first dosage of the Pfizer-BioNTech vaccine, Bril et al. reported the first case of liver injury (Bril et al., 2021). Since then, a series of AEFI have been reported (Shroff et al., 2022). A worldwide case series study has shown that SARS-CoV-2 vaccinations (Pfizer-BioNTech, Moderna, and Oxford–AstraZeneca) have the potential to be hepatotoxic (Efe et al., 2022). Liver injury was predominantly hepatocellular and showed features of immune-mediated hepatitis (Efe et al., 2022). Administering vaccines to individuals with cancer during immunotherapy and with a history of hepatitis C virus (HCV) resulted in a higher risk of autoimmune hepatitis after the administration of the COVID-19 vaccine (Hasegawa et al., 2022; Lasagna et al., 2022). Although the exact mechanism of SARS-CoV-2 vaccine-induced liver injury is still unknown, published reports of SARS-CoV-2 vaccine-induced liver injury predominantly refer to hepatocellular issues frequently characterized by autoimmune hepatitis-like symptoms (Bril et al., 2021; Efe et al., 2022; Hasegawa et al., 2022; Lasagna et al., 2022).

Interestingly, at least six cases of gross hematuria have been reported in patients with a history of biopsy-proven IgA nephropathy (IgAN), involving both mRNA vaccines (Negrea and Rovin, 2021; Perrin et al., 2021; Rahim et al., 2021). All of the previous patients were treated with supportive therapy, and there was rapid resolution of hematuria and no AKI. There were also patients who had prior biopsy-proven IgAN, who developed gross hematuria after their second dose of the BNT162b2 vaccine, without a preceding COVID-19 infection, and patients who had minimal change in disease and AKI following BNT162b2 vaccination (D’agati et al., 2021; Plasse et al., 2021). After receiving the first dose of the BNT162b2 COVID-19 vaccine, a 20-year-old male college student developed AKI that required hemodialysis (Kahn et al., 2021). According to Luo et al.’s statement in an epidemiological study, mRNA vaccinations (such as BioNTech and mRNA-1273) were more frequently linked to AKI than adenovirus vaccines (such as Ad26.COV2.S), which was consistent with our results (Luo et al., 2022). Although mRNA vaccine technology has been developed and improved for nearly 20 years, it has not been widely used in clinical practice until recently (Pardi et al., 2020). There is no convincing hypothesis for the mechanism of SARS-CoV-2 vaccine-induced AKI yet, but it should be noted that there are potential cross-reactivity risks between autoimmune target proteins and SARS-CoV-2, which could lead to immune activation and trigger autoimmune diseases (Segal and Shoenfeld, 2018; Vojdani and Kharrazian, 2020)**.** No AKI events were reported to be associated with the Ad26.COV2.S COVID-19 vaccine; perhaps this vaccine is a better choice for patients with a history of kidney disease.

There is little literature on vaccine association with heart failure and arrhythmias. For example, there was a case of a patient who developed hyperthyroidism complicated with atrial fibrillation and heart failure on the sixth day after the first dose of BNT162b2 (Yamamoto et al., 2021). Tachycardia and hypertension were common adverse events observed with vaccines in a study based on the WHO database, with an incidence rate of 16.41% and 5.82%, respectively (Jeet Kaur et al., 2021). Furthermore, a healthy vaccine recipient was diagnosed with postural orthostatic tachycardia (Reddy et al., 2021) on the sixth day after he received the first dose of BNT162B2. The mechanism for the AEFI of the cardiovascular system associated with COVID-19 vaccination remains unknown and is possibly caused by an immunological response to adrenergic receptors that impair vasoconstriction, which therefore leads to postural tachycardia (Li et al., 2014; Reddy et al., 2021). Fewer heart failure events were reported to be associated with Ad26.COV2.S; perhaps this vaccine is more appropriate for patients with a history of heart failure. Seizure events only showed association with BNT162b2 and Ad26.COV2.S. For patients with a history of epilepsy, the mRNA-1273 vaccine is a better option.

### 4.4 Limitations of the study

It is important to recognize that there are limitations to the data mining procedure using the VAERS database. First, because of the feature of the spontaneous reporting system that the total number of patients vaccinated was unknown, the prevalence of AEFI associated with COVID-19 vaccines could not be calculated. For example, since it is not possible to determine the proportion of pregnant women who received the three vaccines in the real world, the incidence of birth defects is merely the statistical tendency using the VAERS data. If the proportion of pregnant women was higher in some vaccines, the incidence of birth defects may increase correspondingly. Second, the causal relationships between AEFI and the suspected vaccines in the AEFI reports submitted to the VAERS could be questionable since the identification or diagnosis of AEFI was based on the experience of health workers and co-morbidities of the patients. Third, the analysis of COVID-related adverse events should be carefully interpreted since the VAERS is specialized to accept adverse event cases associated with vaccination, and there was hardly any information about indications for the vaccine recipients or whether the vaccine recipients had ever been infected with COVID-19 before the vaccination. Fourth, cases possibly resulting in death were the percentage of cases that ended with fatal outcomes among all the cases where individuals were vaccinated with the same vaccines. It was unknown at the time if they were related, and there was no further investigation. Fifth, we did not analyze the symptom text provided by VAERSDATA. The text provided us with the adverse event in detail based on “symptom,” which is an abstract of symptom text; however, the spontaneous reporting system still demonstrated enlightening clues regarding how COVID-19 vaccines are associated with a higher risk of AEFI from the data mining and signal detection point of view.

## 5 Conclusion

In conclusion, a disproportionality analysis was performed using the ROR method based on the VAERS data. Ad26.COV2.S vaccination was associated with a lower death frequency than BNT162b2 and mRNA-1273. Ad26.COV2.S vaccination was associated with a lower birth defect and emergency room visit frequency than BNT162b2. A minor proportion of vaccine recipients suffered from COVID-19-related symptoms after vaccination with the three vaccines but not as a causal relationship. Thrombotic, hemorrhage, thrombocytopenia, cardiac arrhythmia, hypertension, and hepatotoxicity events were associated with the COVID-19 vaccines BNT162b2, Ad26.COV2.S, and mRNA-1273. Cardiac failure events and acute renal impairment events showed associations with BNT162b2 and mRNA-1273. Seizure events were all associated with BNT162b2 and Ad26.COV2.S. Patients with underlying medical conditions may be advised to use the aforementioned information for vaccination selection. These results are consistent with those of previous studies. The risk/benefit profile of these vaccines remains unchanged, although it is important to maintain vigilance.

## Data Availability

Publicly available datasets were analyzed in this study. These data can be found here: https://vaers.hhs.gov/.
