# Supplementary figures and images for: Serious adverse reaction associated with the COVID-19 vaccines of BNT162b2, Ad26.COV2.S, and mRNA-1273: Gaining insight through the VAERS
Source: Front Pharmacol. 2022 Nov 7;13:921760. doi: 10.3389/fphar.2022.921760 (PMC9676979; doi:10.3389/fphar.2022.921760)

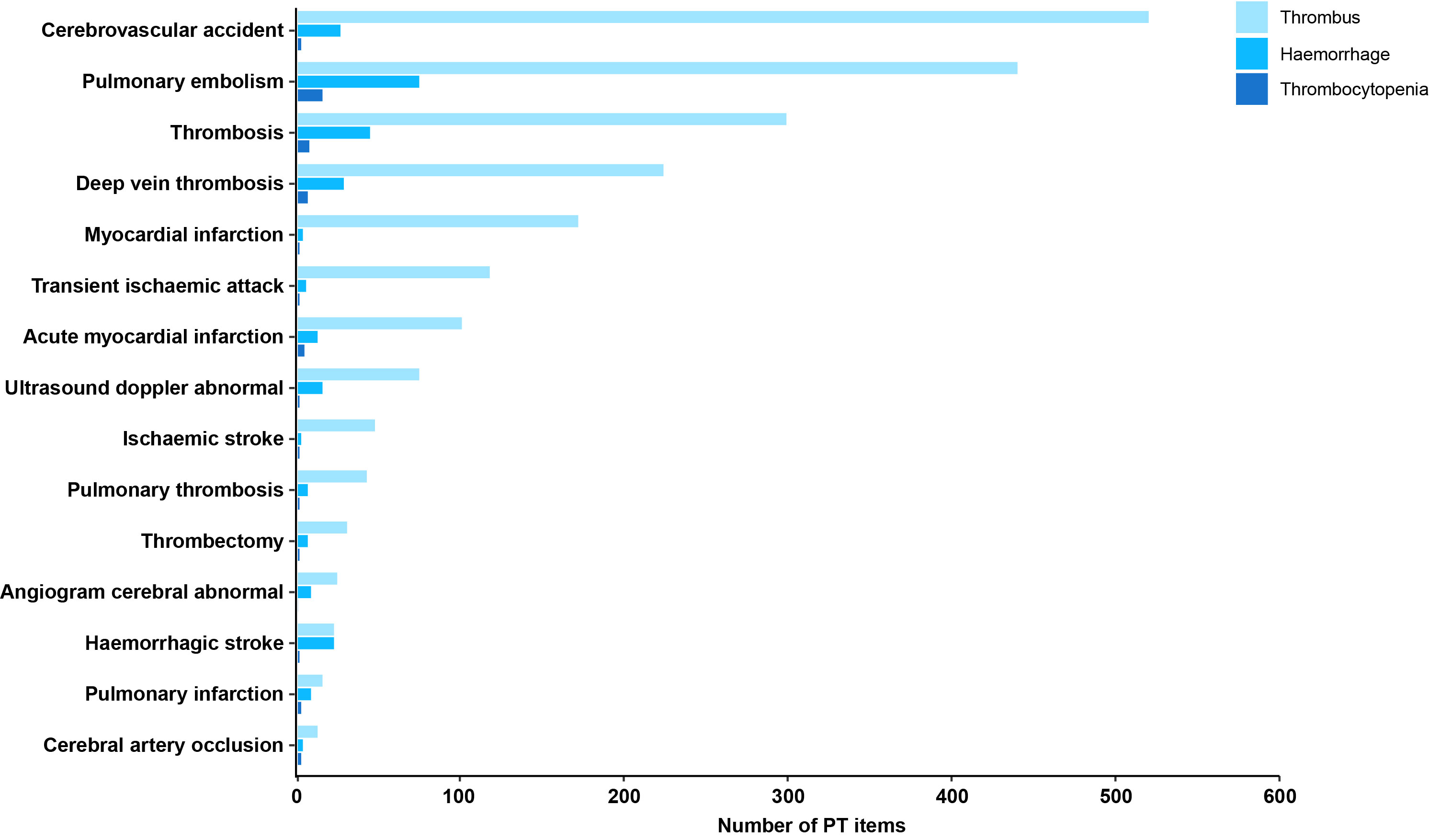

Supplement: Supplementary file 1 [file Image3.JPEG]

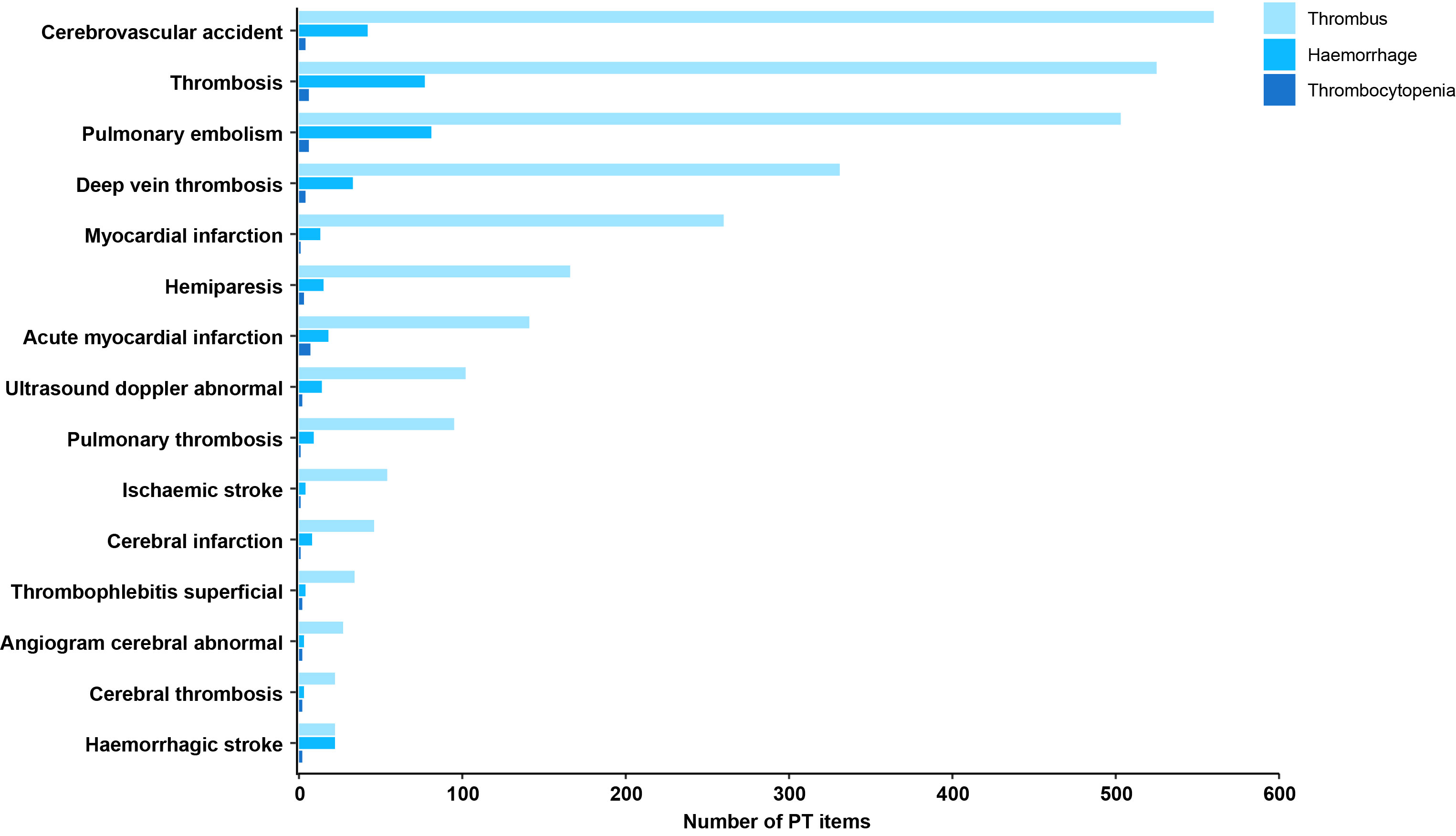

Supplement: Supplementary file 6 [file Image1.JPEG]

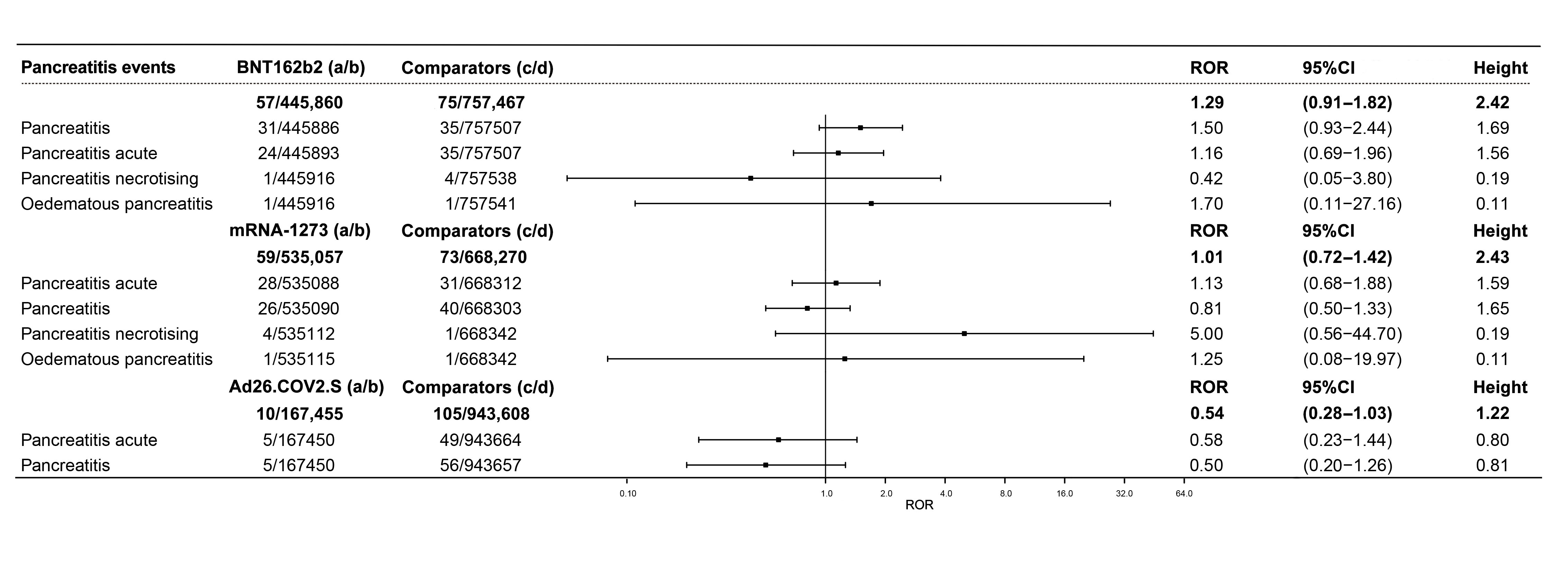

Supplement: Supplementary file 7 [file Image4.JPEG]

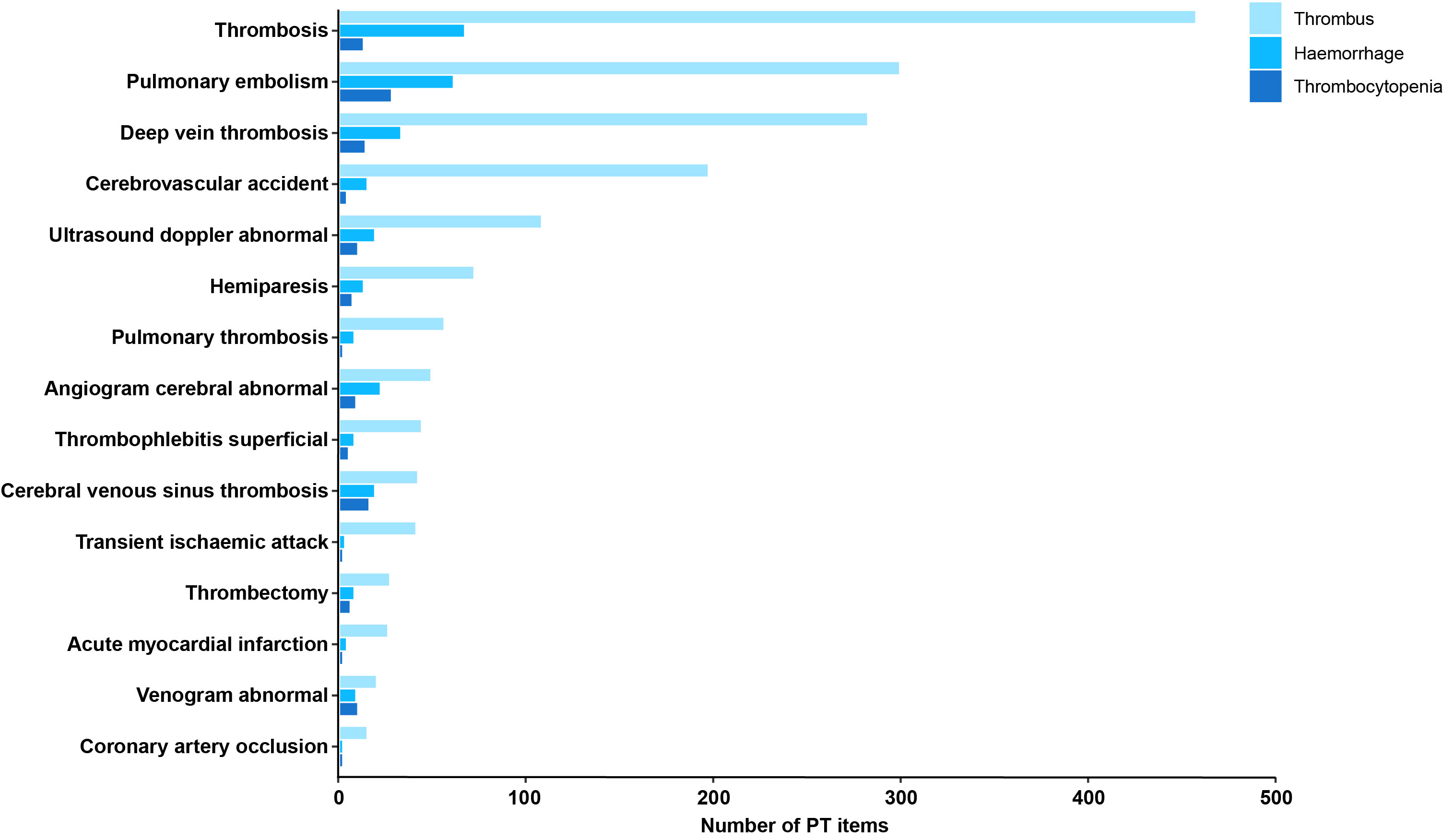

Supplement: Supplementary file 9 [file Image2.JPEG]

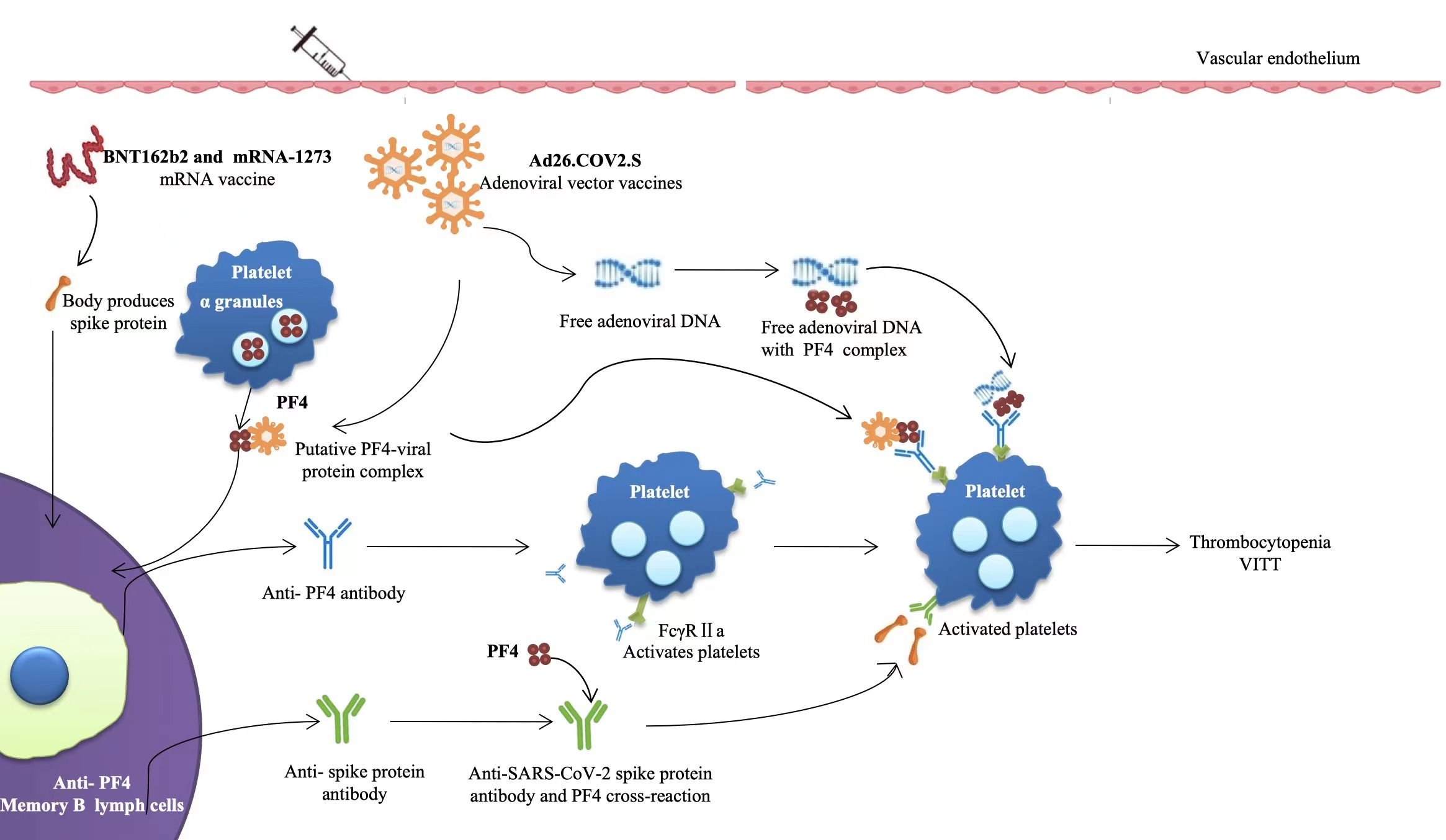

Supplement: Supplementary file 10 [file Image5.JPEG]
