# Supplementary material for: Serious adverse reaction associated with the COVID-19 vaccines of BNT162b2, Ad26.COV2.S, and mRNA-1273: Gaining insight through the VAERS
Source: Front Pharmacol. 2022 Nov 7;13:921760. doi: 10.3389/fphar.2022.921760 (PMC9676979; doi:10.3389/fphar.2022.921760)
Supplement: Supplementary file 2 [file Table1.DOCX]

Table 1 The severe AEFI associated with the BNT162b2, Ad26.COV2.S, and mRNA-1273 vaccines.

| **Severe AEFIs** | **BNT162b2** | **Ad26.COV2.S** | **mRNA-1273** |
| --- | --- | --- | --- |
| Covid events | Yes | Yes | Yes |
| Thrombotic events | Yes | Yes | Yes |
| Haemorrhage events | Yes | Yes | Yes |
| Thrombocytopenia events | Yes | Yes | Yes |
| Cardiac arrthythmias events | Yes | Yes | Yes |
| Cardiac failure events | Yes | Yes * | Yes |
| Hypertension events | Yes | Yes | Yes |
| Hepatotoxicity events | Yes | Yes | Yes |
| Acute renal impairment events | Yes | / | Yes |
| Seizures events | Yes | Yes | / |
| Pancreatitis events | / | / | / |

* Only 15 cases of cardiac failure events associated with the Ad26.COV2.S vaccines.
