# Supplementary material for: Serious adverse reaction associated with the COVID-19 vaccines of BNT162b2, Ad26.COV2.S, and mRNA-1273: Gaining insight through the VAERS
Source: Front Pharmacol. 2022 Nov 7;13:921760. doi: 10.3389/fphar.2022.921760 (PMC9676979; doi:10.3389/fphar.2022.921760)
Supplement: Supplementary file 3 [file Table15.DOCX]

Supplementary Table 14 The median value of time from the vaccination to the onset (TTO) referring to the COVID-19 vaccines of BNT162b2, Ad26.COV2.S, and mRNA-1273.

| **Severe AEFI** | **BNT162b** | **Ad26.COV2.S** | **mRNA-1273** |
| --- | --- | --- | --- |
| Covid events | 150.00 (49.00, 220.00) | 150.00 (62.00, 221.00) | 156.00 (33.00, 229.00) |
| Thrombotic events | 9.00 (2.00, 33.00) | 13.00 (4.00, 31.00) | 10.00 (2.00, 33.00) |
| Haemorrhage events | 2.00 (1.00, 14.00) | 4.00 (1.00, 19.00) | 2.00 (1.00, 14.00) |
| Thrombocytopenia events | 11.00 (2.00, 39.00) | 13.00 (6.00, 30.50) | 11.00 (2.00, 35.50) |
| Cardio Arrhythmias events | 0.00 (0.00, 2.00) | 0.00 (0.00, 1.00) | 0.00 (0.00, 3.00) |
| Cardio Failure events | 1.00 (0.00, 10.00) | 5.00 (1.00, 22.00) | 2.00 (0.00, 9.00) |
| Hypertension events | 0.00 (0.00, 3.00) | 1.00 (0.00, 9.00) | 0.00 (0.00, 5.00) |
| Hepatotoxicity events | 8.00 (1.00, 64.00) | 10.00 (2.00, 58.50) | 8.00 (1.00, 56.00) |
| Acute Renal Impairment events | 57.00 (9.00, 183.00) | 119.00 (13.00, 220.00) | 57.50 (6.00, 204.75) |
| Seizures events | 0.00 (0.00, 2.00) | 0.00 (0.00, 1.00) | 1.00 (0.00, 3.00) |
| Pancreatitis events | 5.00 (1.00, 14.00) | 16.00 (5.00, 127.00) | 7.00 (1.00, 29.50) |

Data are shown as mean ± standard deviation
