# Supplementary material for: Serious adverse reaction associated with the COVID-19 vaccines of BNT162b2, Ad26.COV2.S, and mRNA-1273: Gaining insight through the VAERS
Source: Front Pharmacol. 2022 Nov 7;13:921760. doi: 10.3389/fphar.2022.921760 (PMC9676979; doi:10.3389/fphar.2022.921760)
Supplement: Supplementary file 4 [file Table8.DOCX]

Supplementary Table 7 The preferred term of hepatotoxicity used in this study.

|  | **Preferred term** | **Code** |
| --- | --- | --- |
| **Drug related hepatic disorders - comprehensive search (SMQ)** | | |
| 1 | Bilirubin excretion disorder | 10061009 |
| 2 | Cholaemia | 10048611 |
| 3 | Cholestasis | 10008635 |
| 4 | Cholestatic liver injury | 10067969 |
| 5 | Cholestatic pruritus | 10064190 |
| 6 | Drug-induced liver injury | 10072268 |
| 7 | Hepatitis cholestatic | 10019754 |
| 8 | Hyperbilirubinaemia | 10020578 |
| 9 | Icterus index increased | 10021209 |
| 10 | Jaundice | 10023126 |
| 11 | Jaundice cholestatic | 10023129 |
| 12 | Jaundice hepatocellular | 10023136 |
| 13 | Mixed liver injury | 10066758 |
| 14 | Ocular icterus | 10058117 |
| 15 | Parenteral nutrition associated liver disease | 10074151 |
| 16 | Deficiency of bile secretion | 10071634 |
| 17 | Yellow skin | 10048245 |
| **Drug related hepatic disorders - severe events only (SMQ)** | | |
| 1 | Acquired hepatocerebral degeneration | 10080860 |
| 2 | Acute hepatic failure | 10000804 |
| 3 | Acute on chronic liver failure | 10077305 |
| 4 | Acute yellow liver atrophy | 10070815 |
| 5 | Ascites | 10003445 |
| 6 | Asterixis | 10003547 |
| 7 | Bacterascites | 10068547 |
| 8 | Biliary cirrhosis | 10004659 |
| 9 | Biliary fibrosis | 10004664 |
| 10 | Cardiohepatic syndrome | 10082480 |
| 11 | Cholestatic liver injury | 10067969 |
| 12 | Chronic hepatic failure | 10057573 |
| 13 | Coma hepatic | 10010075 |
| 14 | Cryptogenic cirrhosis | 10063075 |
| 15 | Diabetic hepatopathy | 10071265 |
| 16 | Drug-induced liver injury | 10072268 |
| 17 | Duodenal varices | 10051010 |
| 18 | Gallbladder varices | 10072319 |
| 19 | Gastric variceal injection | 10076237 |
| 20 | Gastric variceal ligation | 10076238 |
| 21 | Gastric varices | 10051012 |
| 22 | Gastric varices haemorrhage | 10057572 |
| 23 | Gastrooesophageal variceal haemorrhage prophylaxis | 10066597 |
| 24 | Hepatectomy | 10061997 |
| 25 | Hepatic atrophy | 10019637 |
| 26 | Hepatic calcification | 10065274 |
| 27 | Hepatic cirrhosis | 10019641 |
| 28 | Hepatic encephalopathy | 10019660 |
| 29 | Hepatic encephalopathy prophylaxis | 10066599 |
| 30 | Hepatic failure | 10019663 |
| 31 | Hepatic fibrosis | 10019668 |
| 32 | Hepatic hydrothorax | 10067365 |
| 33 | Hepatic infiltration eosinophilic | 10064668 |
| 34 | Hepatic lesion | 10061998 |
| 35 | Hepatic necrosis | 10019692 |
| 36 | Hepatic steato-fibrosis | 10077215 |
| 37 | Hepatic steatosis | 10019708 |
| 38 | Hepatitis fulminant | 10019772 |
| 39 | Hepatobiliary disease | 10062000 |
| 40 | Hepatocellular foamy cell syndrome | 10053244 |
| 41 | Hepatocellular injury | 10019837 |
| 42 | Hepatopulmonary syndrome | 10052274 |
| 43 | Hepatorenal failure | 10019845 |
| 44 | Hepatorenal syndrome | 10019846 |
| 45 | Hepatotoxicity | 10019851 |
| 46 | Immune-mediated cholangitis | 10083406 |
| 47 | Immune-mediated hepatic disorder | 10083521 |
| 48 | Intestinal varices | 10071502 |
| 49 | Intestinal varices haemorrhage | 10078058 |
| 50 | Liver dialysis | 10076640 |
| 51 | Liver disorder | 10024670 |
| 52 | Liver injury | 10067125 |
| 53 | Liver operation | 10062040 |
| 54 | Liver transplant | 10024714 |
| 55 | Lupoid hepatic cirrhosis | 10025129 |
| 56 | Minimal hepatic encephalopathy | 10076204 |
| 57 | Mixed liver injury | 10066758 |
| 58 | Nodular regenerative hyperplasia | 10051081 |
| 59 | Nonalcoholic fatty liver disease | 10082249 |
| 60 | Non-alcoholic steatohepatitis | 10053219 |
| 61 | Non-cirrhotic portal hypertension | 10077259 |
| 62 | Oedema due to hepatic disease | 10049631 |
| 63 | Oesophageal varices haemorrhage | 10030210 |
| 64 | Peripancreatic varices | 10073215 |
| 65 | Portal fibrosis | 10074726 |
| 66 | Portal hypertension | 10036200 |
| 67 | Portal hypertensive colopathy | 10079446 |
| 68 | Portal hypertensive enteropathy | 10068923 |
| 69 | Portal hypertensive gastropathy | 10050897 |
| 70 | Portal vein cavernous transformation | 10073979 |
| 71 | Portal vein dilatation | 10073209 |
| 72 | Portopulmonary hypertension | 10067281 |
| 73 | Primary biliary cholangitis | 10080429 |
| 74 | Regenerative siderotic hepatic nodule | 10080679 |
| 75 | Renal and liver transplant | 10052279 |
| 76 | Retrograde portal vein flow | 10067338 |
| 77 | Reye's syndrome | 10039012 |
| 78 | Reynold's syndrome | 10070953 |
| 79 | Splenic varices | 10067823 |
| 80 | Splenic varices haemorrhage | 10068662 |
| 81 | Steatohepatitis | 10076331 |
| 82 | Subacute hepatic failure | 10056956 |
| 83 | Sugiura procedure | 10083010 |
| 84 | Varices oesophageal | 10056091 |
| 85 | Varicose veins of abdominal wall | 10072284 |
| 86 | White nipple sign | 10078438 |
| 87 | Anorectal varices | 10068924 |
| 88 | Anorectal varices haemorrhage | 10068925 |
| 89 | Complications of transplanted liver | 10010186 |
| 90 | Hepatic perfusion disorder | 10083840 |
| 91 | Increased liver stiffness | 10082444 |
| 92 | Intrahepatic portal hepatic venous fistula | 10072629 |
| 93 | Liver and pancreas transplant rejection | 10051603 |
| 94 | Liver contusion | 10067266 |
| 95 | Liver transplant failure | 10083175 |
| 96 | Liver transplant rejection | 10024715 |
| 97 | Multivisceral transplantation | 10082450 |
| 98 | Peritoneovenous shunt | 10052716 |
| 99 | Portal shunt | 10036204 |
| 100 | Portal shunt procedure | 10077479 |
| 101 | Small-for-size liver syndrome | 10069380 |
| 102 | Spider naevus | 10041519 |
| 103 | Splenic artery embolisation | 10083795 |
| 104 | Splenorenal shunt | 10041661 |
| 105 | Splenorenal shunt procedure | 10077281 |
| 106 | Spontaneous intrahepatic portosystemic venous shunt | 10076239 |
| 107 | Stomal varices | 10075186 |
| **Hepatitis, non-infectious (SMQ)** | | |
| 1 | Acute graft versus host disease in liver | 10066263 |
| 2 | Allergic hepatitis | 10071198 |
| 3 | Alloimmune hepatitis | 10080576 |
| 4 | Autoimmune hepatitis | 10003827 |
| 5 | Chronic graft versus host disease in liver | 10072160 |
| 6 | Chronic hepatitis | 10008909 |
| 7 | Graft versus host disease in liver | 10064676 |
| 8 | Hepatitis | 10019717 |
| 9 | Hepatitis acute | 10019727 |
| 10 | Hepatitis cholestatic | 10019754 |
| 11 | Hepatitis chronic active | 10019755 |
| 12 | Hepatitis chronic persistent | 10019759 |
| 13 | Hepatitis fulminant | 10019772 |
| 14 | Hepatitis toxic | 10019795 |
| 15 | Immune-mediated hepatitis | 10078962 |
| 16 | Ischaemic hepatitis | 10023025 |
| 17 | Lupus hepatitis | 10067737 |
| 18 | Non-alcoholic steatohepatitis | 10053219 |
| 19 | Radiation hepatitis | 10051015 |
| 20 | Steatohepatitis | 10076331 |
| 21 | Granulomatous liver disease | 10018704 |
| 22 | Liver sarcoidosis | 10068664 |
| 23 | Portal tract inflammation | 10075331 |
| **Liver neoplasms, benign (incl cysts and polyps) (SMQ)** | | |
| 1 | Benign hepatic neoplasm | 10004269 |
| 2 | Benign hepatobiliary neoplasm | 10077922 |
| 3 | Focal nodular hyperplasia | 10052285 |
| 4 | Haemangioma of liver | 10018821 |
| 5 | Haemorrhagic hepatic cyst | 10067796 |
| 6 | Hepatic adenoma | 10019629 |
| 7 | Hepatic cyst | 10019646 |
| 8 | Hepatic cyst ruptured | 10053973 |
| 9 | Hepatic haemangioma rupture | 10054885 |
| 10 | Hepatic hamartoma | 10079685 |
| 11 | Hepatobiliary cyst | 10079889 |
| **Liver neoplasms, malignant and unspecified (SMQ)** | | |
| 1 | Cholangiosarcoma | 10077861 |
| 2 | Hepatic angiosarcoma | 10067388 |
| 3 | Hepatic cancer | 10073069 |
| 4 | Hepatic cancer metastatic | 10055110 |
| 5 | Hepatic cancer recurrent | 10073070 |
| 6 | Hepatic cancer stage I | 10059318 |
| 7 | Hepatic cancer stage II | 10059319 |
| 8 | Hepatic cancer stage III | 10059324 |
| 9 | Hepatic cancer stage IV | 10059325 |
| 10 | Hepatobiliary cancer | 10073073 |
| 11 | Hepatobiliary cancer in situ | 10073074 |
| 12 | Hepatoblastoma | 10062001 |
| 13 | Hepatoblastoma recurrent | 10019823 |
| 14 | Hepatocellular carcinoma | 10073071 |
| 15 | Liver carcinoma ruptured | 10050842 |
| 16 | Mixed hepatocellular cholangiocarcinoma | 10027761 |
| 17 | Liver ablation | 10074766 |
| 18 | Hepatic neoplasm | 10019695 |
| 19 | Hepatobiliary neoplasm | 10061203 |
| **Liver related investigations, signs and symptoms (SMQ)** | | |
| 1 | Alanine aminotransferase abnormal | 10001547 |
| 2 | Alanine aminotransferase increased | 10001551 |
| 3 | Ammonia abnormal | 10001942 |
| 4 | Ammonia increased | 10001946 |
| 5 | Ascites | 10003445 |
| 6 | Aspartate aminotransferase abnormal | 10003477 |
| 7 | Aspartate aminotransferase increased | 10003481 |
| 8 | AST/ALT ratio abnormal | 10082832 |
| 9 | Bacterascites | 10068547 |
| 10 | Bile output abnormal | 10051344 |
| 11 | Bile output decreased | 10051343 |
| 12 | Biliary ascites | 10074150 |
| 13 | Bilirubin conjugated abnormal | 10067718 |
| 14 | Bilirubin conjugated increased | 10004685 |
| 15 | Bilirubin urine present | 10077356 |
| 16 | Biopsy liver abnormal | 10004792 |
| 17 | Blood bilirubin abnormal | 10058477 |
| 18 | Blood bilirubin increased | 10005364 |
| 19 | Blood bilirubin unconjugated increased | 10005370 |
| 20 | Bromosulphthalein test abnormal | 10006408 |
| 21 | Child-Pugh-Turcotte score abnormal | 10077020 |
| 22 | Child-Pugh-Turcotte score increased | 10068287 |
| 23 | Computerised tomogram liver | 10076215 |
| 24 | Computerised tomogram liver abnormal | 10078360 |
| 25 | Congestive hepatopathy | 10084058 |
| 26 | Foetor hepaticus | 10052554 |
| 27 | Galactose elimination capacity test abnormal | 10059710 |
| 28 | Galactose elimination capacity test decreased | 10059712 |
| 29 | Gamma-glutamyltransferase abnormal | 10017688 |
| 30 | Gamma-glutamyltransferase increased | 10017693 |
| 31 | Guanase increased | 10051333 |
| 32 | Hepaplastin abnormal | 10019621 |
| 33 | Hepaplastin decreased | 10019622 |
| 34 | Hepatic artery flow decreased | 10068997 |
| 35 | Hepatic enzyme abnormal | 10062685 |
| 36 | Hepatic enzyme decreased | 10060794 |
| 37 | Hepatic enzyme increased | 10060795 |
| 38 | Hepatic function abnormal | 10019670 |
| 39 | Hepatic hydrothorax | 10067365 |
| 40 | Hepatic hypertrophy | 10076254 |
| 41 | Hepatic mass | 10057110 |
| 42 | Hepatic pain | 10019705 |
| 43 | Hepatic sequestration | 10066244 |
| 44 | Hepatic vascular resistance increased | 10068358 |
| 45 | Hepatic venous pressure gradient abnormal | 10083172 |
| 46 | Hepatic venous pressure gradient increased | 10083171 |
| 47 | Hepatobiliary scan abnormal | 10066195 |
| 48 | Hepatomegaly | 10019842 |
| 49 | Hepatosplenomegaly | 10019847 |
| 50 | Hyperammonaemia | 10020575 |
| 51 | Hyperbilirubinaemia | 10020578 |
| 52 | Hypercholia | 10051924 |
| 53 | Hypertransaminasaemia | 10068237 |
| 54 | Kayser-Fleischer ring | 10023321 |
| 55 | Liver function test abnormal | 10024690 |
| 56 | Liver function test decreased | 10077677 |
| 57 | Liver function test increased | 10077692 |
| 58 | Liver induration | 10052550 |
| 59 | Liver palpable | 10075895 |
| 60 | Liver scan abnormal | 10061947 |
| 61 | Liver tenderness | 10024712 |
| 62 | Magnetic resonance imaging liver abnormal | 10083123 |
| 63 | Magnetic resonance proton density fat fraction measurement | 10082443 |
| 64 | Mitochondrial aspartate aminotransferase increased | 10064712 |
| 65 | Molar ratio of total branched-chain amino acid to tyrosine | 10066869 |
| 66 | Oedema due to hepatic disease | 10049631 |
| 67 | Perihepatic discomfort | 10054125 |
| 68 | Retrograde portal vein flow | 10067338 |
| 69 | Total bile acids increased | 10064558 |
| 70 | Transaminases abnormal | 10062688 |
| 71 | Transaminases increased | 10054889 |
| 72 | Ultrasound liver abnormal | 10045428 |
| 73 | Urine bilirubin increased | 10050792 |
| 74 | White nipple sign | 10078438 |
| 75 | X-ray hepatobiliary abnormal | 10056536 |
| 76 | 5'nucleotidase increased | 10000028 |
| 77 | Blood alkaline phosphatase abnormal | 10059571 |
| 78 | Blood alkaline phosphatase increased | 10059570 |
| 79 | Blood cholinesterase abnormal | 10005429 |
| 80 | Blood cholinesterase decreased | 10005430 |
| 81 | Deficiency of bile secretion | 10071634 |
| 82 | Glutamate dehydrogenase increased | 10049483 |
| 83 | Glycocholic acid increased | 10080824 |
| 84 | Haemorrhagic ascites | 10059766 |
| 85 | Hepatic fibrosis marker abnormal | 10074084 |
| 86 | Hepatic fibrosis marker increased | 10074413 |
| 87 | Hepatic lymphocytic infiltration | 10079686 |
| 88 | Hypoalbuminaemia | 10020942 |
| 89 | Leucine aminopeptidase increased | 10024275 |
| 90 | Liver iron concentration abnormal | 10074352 |
| 91 | Liver iron concentration increased | 10074354 |
| 92 | Liver opacity | 10084071 |
| 93 | Model for end stage liver disease score abnormal | 10077291 |
| 94 | Model for end stage liver disease score increased | 10077292 |
| 95 | Periportal oedema | 10068821 |
| 96 | Peritoneal fluid protein abnormal | 10069000 |
| 97 | Peritoneal fluid protein decreased | 10068999 |
| 98 | Peritoneal fluid protein increased | 10068998 |
| 99 | Pneumobilia | 10066004 |
| 100 | Portal vein flow decreased | 10067337 |
| 101 | Portal vein pressure increased | 10064936 |
| 102 | Retinol binding protein decreased | 10048473 |
| 103 | Urobilinogen urine decreased | 10070480 |
| 104 | Urobilinogen urine increased | 10070479 |
| **Liver-related coagulation and bleeding disturbances (SMQ)** | | |
| 1 | Acquired antithrombin III deficiency | 10074561 |
| 2 | Acquired factor IX deficiency | 10082747 |
| 3 | Acquired factor VIII deficiency | 10082745 |
| 4 | Acquired factor XI deficiency | 10082746 |
| 5 | Acquired protein S deficiency | 10068370 |
| 6 | Anti factor X activity abnormal | 10077670 |
| 7 | Anti factor X activity decreased | 10077674 |
| 8 | Anti factor X activity increased | 10077671 |
| 9 | Antithrombin III decreased | 10049547 |
| 10 | Blood fibrinogen abnormal | 10005518 |
| 11 | Blood fibrinogen decreased | 10005520 |
| 12 | Blood thrombin abnormal | 10005818 |
| 13 | Blood thrombin decreased | 10005820 |
| 14 | Blood thromboplastin abnormal | 10005824 |
| 15 | Blood thromboplastin decreased | 10005826 |
| 16 | Coagulation factor decreased | 10009736 |
| 17 | Coagulation factor IX level abnormal | 10061770 |
| 18 | Coagulation factor IX level decreased | 10009746 |
| 19 | Coagulation factor V level abnormal | 10061771 |
| 20 | Coagulation factor V level decreased | 10009754 |
| 21 | Coagulation factor VII level abnormal | 10061772 |
| 22 | Coagulation factor VII level decreased | 10009761 |
| 23 | Coagulation factor X level abnormal | 10061774 |
| 24 | Coagulation factor X level decreased | 10009775 |
| 25 | Hyperfibrinolysis | 10074737 |
| 26 | Hypocoagulable state | 10020973 |
| 27 | Hypofibrinogenaemia | 10051125 |
| 28 | Hypoprothrombinaemia | 10021085 |
| 29 | Hypothrombinaemia | 10058517 |
| 30 | Hypothromboplastinaemia | 10058518 |
| 31 | International normalised ratio abnormal | 10022592 |
| 32 | International normalised ratio increased | 10022595 |
| 33 | Protein C decreased | 10037005 |
| 34 | Protein S abnormal | 10051736 |
| 35 | Protein S decreased | 10051120 |
| 36 | Prothrombin level abnormal | 10037048 |
| 37 | Prothrombin level decreased | 10037050 |
| 38 | Prothrombin time abnormal | 10037057 |
| 39 | Prothrombin time prolonged | 10037063 |
| 40 | Prothrombin time ratio abnormal | 10061918 |
| 41 | Prothrombin time ratio increased | 10037068 |
| 42 | Thrombin time abnormal | 10051319 |
| 43 | Thrombin time prolonged | 10051390 |
| 44 | International normalised ratio decreased | 10022594 |
| 45 | Prothrombin time ratio decreased | 10037066 |
| 98 | Peritoneal fluid protein increased | 10068998 |
| 99 | Pneumobilia | 10066004 |
| 100 | Portal vein flow decreased | 10067337 |
| 101 | Portal vein pressure increased | 10064936 |
| 102 | Retinol binding protein decreased | 10048473 |
| 103 | Urobilinogen urine decreased | 10070480 |
| 104 | Urobilinogen urine increased | 10070479 |
| **Liver-related coagulation and bleeding disturbances (SMQ)** | | |
| 1 | Acquired antithrombin III deficiency | 10074561 |
| 2 | Acquired factor IX deficiency | 10082747 |
| 3 | Acquired factor VIII deficiency | 10082745 |
| 4 | Acquired factor XI deficiency | 10082746 |
| 5 | Acquired protein S deficiency | 10068370 |
| 6 | Anti factor X activity abnormal | 10077670 |
| 7 | Anti factor X activity decreased | 10077674 |
| 8 | Anti factor X activity increased | 10077671 |
| 9 | Antithrombin III decreased | 10049547 |
| 10 | Blood fibrinogen abnormal | 10005518 |
| 11 | Blood fibrinogen decreased | 10005520 |
| 12 | Blood thrombin abnormal | 10005818 |
| 13 | Blood thrombin decreased | 10005820 |
| 14 | Blood thromboplastin abnormal | 10005824 |
| 15 | Blood thromboplastin decreased | 10005826 |
| 16 | Coagulation factor decreased | 10009736 |
| 17 | Coagulation factor IX level abnormal | 10061770 |
| 18 | Coagulation factor IX level decreased | 10009746 |
| 19 | Coagulation factor V level abnormal | 10061771 |
| 20 | Coagulation factor V level decreased | 10009754 |
| 21 | Coagulation factor VII level abnormal | 10061772 |
| 22 | Coagulation factor VII level decreased | 10009761 |
| 23 | Coagulation factor X level abnormal | 10061774 |
| 24 | Coagulation factor X level decreased | 10009775 |
| 25 | Hyperfibrinolysis | 10074737 |
| 26 | Hypocoagulable state | 10020973 |
| 27 | Hypofibrinogenaemia | 10051125 |
| 28 | Hypoprothrombinaemia | 10021085 |
| 29 | Hypothrombinaemia | 10058517 |
| 30 | Hypothromboplastinaemia | 10058518 |
| 31 | International normalised ratio abnormal | 10022592 |
| 32 | International normalised ratio increased | 10022595 |
| 33 | Protein C decreased | 10037005 |
| 34 | Protein S abnormal | 10051736 |
| 35 | Protein S decreased | 10051120 |
| 36 | Prothrombin level abnormal | 10037048 |
| 37 | Prothrombin level decreased | 10037050 |
| 38 | Prothrombin time abnormal | 10037057 |
| 39 | Prothrombin time prolonged | 10037063 |
| 40 | Prothrombin time ratio abnormal | 10061918 |
| 41 | Prothrombin time ratio increased | 10037068 |
| 42 | Thrombin time abnormal | 10051319 |
| 43 | Thrombin time prolonged | 10051390 |
| 44 | International normalised ratio decreased | 10022594 |
| 45 | Prothrombin time ratio decreased | 10037066 |
