# Supplementary material for: Serious adverse reaction associated with the COVID-19 vaccines of BNT162b2, Ad26.COV2.S, and mRNA-1273: Gaining insight through the VAERS
Source: Front Pharmacol. 2022 Nov 7;13:921760. doi: 10.3389/fphar.2022.921760 (PMC9676979; doi:10.3389/fphar.2022.921760)
Supplement: Supplementary file 5 [file Table12.DOCX]

Supplementary Table 11 Serious adverse events and main complications following immunization associated with the COVID-19 vaccines of BNT162b2 (Pfizer/ BioNTech).

| Died after vaccination (1,963 cases) | Cases | Percents |
| --- | --- | --- |
| Death | 1,579 | 80.44% |
| Dyspnoea | 245 | 12.48% |
| Cardiac arrest | 167 | 8.51% |
| Unresponsive to stimuli | 152 | 7.74% |
| Covid-19 | 148 | 7.54% |
| Sars-Cov-2 test positive | 140 | 7.13% |
| Resuscitation | 122 | 6.21% |
| Asthenia | 113 | 5.76% |
| Vomiting | 110 | 5.60% |
| Fatigue | 104 | 5.30% |
| Threatens life (2,221 cases) | Cases | Percents |
| Dyspnoea | 426 | 19.18% |
| Pulmonary embolism | 262 | 11.80% |
| Headache | 207 | 9.32% |
| Sars-cov-2 test | 196 | 8.82% |
| Blood test | 196 | 8.82% |
| Pyrexia | 193 | 8.69% |
| Cerebrovascular accident | 184 | 8.28% |
| Dizziness | 178 | 8.01% |
| Computerised tomogram | 175 | 7.88% |
| Chest pain | 173 | 7.79% |
| Emergency visit (16,737 cases) | Cases | Percents |
| Dizziness | 3,063 | 18.30% |
| Dyspnoea | 2,606 | 15.57% |
| Headache | 2,203 | 13.16% |
| Nausea | 2,013 | 12.03% |
| Fatigue | 1,471 | 8.79% |
| Pyrexia | 1,305 | 7.80% |
| Pain | 1,274 | 7.61% |
| Chills | 1,189 | 7.10% |
| Chest discomfort | 1,120 | 6.69% |
| Electrocardiogram | 1,110 | 6.63% |
| Hospitalization (7,151 cases) | Cases | Percents |
| Dyspnoea | 1,133 | 15.84% |
| Pyrexia | 739 | 10.33% |
| Sars-cov-2 test | 617 | 8.63% |
| Headache | 610 | 8.53% |
| Fatigue | 571 | 7.98% |
| Nausea | 554 | 7.75% |
| Covid-19 | 551 | 7.71% |
| Chest pain | 543 | 7.59% |
| Dizziness | 530 | 7.41% |
| Computerised tomogram | 527 | 7.37% |

For people received BNT162b2, 1,963 people died after the vaccine (4.40 per 1,000, 1,963/445,926), 2,221 people (4.98 per 1,000, 2,221/445,926) suffered from life-threatening AEFI, and people visited emergency rooms and hospitalization were 16,737 (37.53 per 1,000, 16,737/445,926) and 7,151 (16.04 per 1,000, 7,151/445,926), respectively.
