# Supplementary material for: Serious adverse reaction associated with the COVID-19 vaccines of BNT162b2, Ad26.COV2.S, and mRNA-1273: Gaining insight through the VAERS
Source: Front Pharmacol. 2022 Nov 7;13:921760. doi: 10.3389/fphar.2022.921760 (PMC9676979; doi:10.3389/fphar.2022.921760)
Supplement: Supplementary file 8 [file Table7.DOCX]

Supplementary Table 6 The preferred term of hypertension used in this study.

|  | **Preferred term** | **Code** |
| --- | --- | --- |
| 1 | Accelerated hypertension | 10000358 |
| 2 | Blood pressure ambulatory increased | 10005732 |
| 3 | Blood pressure diastolic increased | 10005739 |
| 4 | Blood pressure inadequately controlled | 10051128 |
| 5 | Blood pressure increased | 10005750 |
| 6 | Blood pressure management | 10063926 |
| 7 | Blood pressure orthostatic increased | 10053355 |
| 8 | Blood pressure systolic increased | 10005760 |
| 9 | Catecholamine crisis | 10081751 |
| 10 | Dialysis induced hypertension | 10063067 |
| 11 | Diastolic hypertension | 10012758 |
| 12 | Eclampsia | 10014129 |
| 13 | Endocrine hypertension | 10057615 |
| 14 | Essential hypertension | 10015488 |
| 15 | Gestational hypertension | 10070538 |
| 16 | HELLP syndrome | 10049058 |
| 17 | Hyperaldosteronism | 10020571 |
| 18 | Hypertension | 10020772 |
| 19 | Hypertension neonatal | 10049781 |
| 20 | Hypertensive angiopathy | 10059238 |
| 21 | Hypertensive cardiomegaly | 10020801 |
| 22 | Hypertensive cardiomyopathy | 10058222 |
| 23 | Hypertensive cerebrovascular disease | 10077000 |
| 24 | Hypertensive crisis | 10020802 |
| 25 | Hypertensive emergency | 10058179 |
| 26 | Hypertensive encephalopathy | 10020803 |
| 27 | Hypertensive end-organ damage | 10079496 |
| 28 | Hypertensive heart disease | 10020823 |
| 29 | Hypertensive nephropathy | 10055171 |
| 30 | Hypertensive urgency | 10058181 |
| 31 | Labile hypertension | 10049079 |
| 32 | Malignant hypertension | 10025600 |
| 33 | Malignant hypertensive heart disease | 10025603 |
| 34 | Malignant renal hypertension | 10026674 |
| 35 | Maternal hypertension affecting foetus | 10026924 |
| 36 | Mean arterial pressure increased | 10026985 |
| 37 | Metabolic syndrome | 10052066 |
| 38 | Neurogenic hypertension | 10067598 |
| 39 | Orthostatic hypertension | 10065508 |
| 40 | Page kidney | 10076704 |
| 41 | Postoperative hypertension | 10050631 |
| 42 | Pre-eclampsia | 10036485 |
| 43 | Prehypertension | 10065918 |
| 44 | Primary hyperaldosteronism | 10036692 |
| 45 | Procedural hypertension | 10062886 |
