# Supplementary material for: Serious adverse reaction associated with the COVID-19 vaccines of BNT162b2, Ad26.COV2.S, and mRNA-1273: Gaining insight through the VAERS
Source: Front Pharmacol. 2022 Nov 7;13:921760. doi: 10.3389/fphar.2022.921760 (PMC9676979; doi:10.3389/fphar.2022.921760)
Supplement: Supplementary file 11 [file Table9.DOCX]

Supplementary Table 8 The preferred term of acute renal impairment used in this study.

|  | **Preferred term** | **Code** |
| --- | --- | --- |
| 1 | Acute kidney injury | 10069339 |
| 2 | Acute phosphate nephropathy | 10069688 |
| 3 | Anuria | 10002847 |
| 4 | Azotaemia | 10003885 |
| 5 | Continuous haemodiafiltration | 10066338 |
| 6 | Dialysis | 10061105 |
| 7 | Foetal renal impairment | 10078987 |
| 8 | Haemodialysis | 10018875 |
| 9 | Haemofiltration | 10053090 |
| 10 | Neonatal anuria | 10049778 |
| 11 | Nephropathy toxic | 10029155 |
| 12 | Oliguria | 10030302 |
| 13 | Peritoneal dialysis | 10034660 |
| 14 | Prerenal failure | 10072370 |
| 15 | Renal failure | 10038435 |
| 16 | Renal failure neonatal | 10038447 |
| 17 | Renal impairment | 10062237 |
| 18 | Renal impairment neonatal | 10049776 |
| 19 | Subacute kidney injury | 10081980 |
