# Supplementary material for: Serious adverse reaction associated with the COVID-19 vaccines of BNT162b2, Ad26.COV2.S, and mRNA-1273: Gaining insight through the VAERS
Source: Front Pharmacol. 2022 Nov 7;13:921760. doi: 10.3389/fphar.2022.921760 (PMC9676979; doi:10.3389/fphar.2022.921760)
Supplement: Supplementary file 12 [file Table2.DOCX]

Supplementary Table 1 The preferred term of thromboembolism used in this study.

| **Arterial embolic and thrombotic events** | **Preferred term** | **Code** |
| --- | --- | --- |
| 1 | Acute aortic syndrome | 10074337 |
| 2 | Acute myocardial infarction | 10000891 |
| 3 | Amaurosis | 10001902 |
| 4 | Amaurosis fugax | 10001903 |
| 5 | Angioplasty | 10002475 |
| 6 | Aortic bypass | 10057617 |
| 7 | Aortic embolus | 10002897 |
| 8 | Aortic surgery | 10061651 |
| 9 | Aortic thrombosis | 10002910 |
| 10 | Aortogram abnormal | 10057794 |
| 11 | Arterectomy | 10071026 |
| 12 | Arterectomy with graft replacement | 10003140 |
| 13 | Arterial angioplasty | 10081731 |
| 14 | Arterial bypass occlusion | 10077766 |
| 15 | Arterial bypass operation | 10056418 |
| 16 | Arterial bypass thrombosis | 10077765 |
| 17 | Arterial graft | 10061655 |
| 18 | Arterial occlusive disease | 10062599 |
| 19 | Arterial stent insertion | 10061657 |
| 20 | Arterial therapeutic procedure | 10052949 |
| 21 | Arterial thrombosis | 10003178 |
| 22 | Arteriogram abnormal | 10061659 |
| 23 | Arteriogram carotid abnormal | 10003195 |
| 24 | Arteriotomy | 10078636 |
| 25 | Atherectomy | 10063025 |
| 26 | Atherosclerotic plaque rupture | 10076604 |
| 27 | Atrial appendage closure | 10079735 |
| 28 | Atrial appendage resection | 10080843 |
| 29 | Basal ganglia infarction | 10069020 |
| 30 | Basilar artery occlusion | 10048963 |
| 31 | Basilar artery thrombosis | 10063093 |
| 32 | Blindness transient | 10005184 |
| 33 | Brachiocephalic artery occlusion | 10069694 |
| 34 | Capsular warning syndrome | 10067744 |
| 35 | Carotid angioplasty | 10071260 |
| 36 | Carotid arterial embolus | 10007684 |
| 37 | Carotid artery bypass | 10053003 |
| 38 | Carotid artery occlusion | 10048964 |
| 39 | Carotid artery stent insertion | 10066102 |
| 40 | Carotid artery thrombosis | 10007688 |
| 41 | Carotid endarterectomy | 10007692 |
| 42 | Cerebellar artery occlusion | 10053633 |
| 43 | Cerebellar artery thrombosis | 10008023 |
| 44 | Cerebellar embolism | 10067167 |
| 45 | Cerebral artery embolism | 10008088 |
| 46 | Cerebral artery occlusion | 10008089 |
| 47 | Cerebral artery stent insertion | 10081893 |
| 48 | Cerebral artery thrombosis | 10008092 |
| 49 | Cerebral hypoperfusion | 10065384 |
| 50 | Cerebral vascular occlusion | 10076895 |
| 51 | Cerebrovascular insufficiency | 10058842 |
| 52 | Cerebrovascular stenosis | 10061751 |
| 53 | Coeliac artery occlusion | 10069696 |
| 54 | Coronary angioplasty | 10050329 |
| 55 | Coronary arterial stent insertion | 10052086 |
| 56 | Coronary artery bypass | 10011077 |
| 57 | Coronary artery embolism | 10011084 |
| 58 | Coronary artery occlusion | 10011086 |
| 59 | Coronary artery reocclusion | 10053261 |
| 60 | Coronary artery surgery | 10011090 |
| 61 | Coronary artery thrombosis | 10011091 |
| 62 | Coronary endarterectomy | 10011101 |
| 63 | Coronary revascularisation | 10049887 |
| 64 | Coronary vascular graft occlusion | 10075162 |
| 65 | Embolia cutis medicamentosa | 10058729 |
| 66 | Embolism | 10061169 |
| 67 | Embolism arterial | 10014513 |
| 68 | Endarterectomy | 10014648 |
| 69 | Femoral artery embolism | 10068365 |
| 70 | Hepatic artery embolism | 10019635 |
| 71 | Hepatic artery occlusion | 10051991 |
| 72 | Hepatic artery thrombosis | 10019636 |
| 73 | Hypothenar hammer syndrome | 10063518 |
| 74 | Iliac artery embolism | 10021338 |
| 75 | Iliac artery occlusion | 10064601 |
| 76 | Internal capsule infarction | 10083408 |
| 77 | Intra-aortic balloon placement | 10052989 |
| 78 | Intraoperative cerebral artery occlusion | 10056382 |
| 79 | Ischaemic cerebral infarction | 10060840 |
| 80 | Ischaemic stroke | 10061256 |
| 81 | Lacunar infarction | 10051078 |
| 82 | Leriche syndrome | 10024242 |
| 83 | Mesenteric arterial occlusion | 10027394 |
| 84 | Mesenteric arteriosclerosis | 10065560 |
| 85 | Mesenteric artery embolism | 10027395 |
| 86 | Mesenteric artery stenosis | 10027396 |
| 87 | Mesenteric artery stent insertion | 10071261 |
| 88 | Mesenteric artery thrombosis | 10027397 |
| 89 | Myocardial infarction | 10028596 |
| 90 | Myocardial necrosis | 10028602 |
| 91 | Ophthalmic artery thrombosis | 10081144 |
| 92 | Papillary muscle infarction | 10033697 |
| 93 | Penile artery occlusion | 10068035 |
| 94 | Percutaneous coronary intervention | 10065608 |
| 95 | Peripheral arterial occlusive disease | 10062585 |
| 96 | Peripheral arterial reocclusion | 10069379 |
| 97 | Peripheral artery angioplasty | 10057518 |
| 98 | Peripheral artery bypass | 10072561 |
| 99 | Peripheral artery occlusion | 10057525 |
| 100 | Peripheral artery stent insertion | 10072562 |
| 101 | Peripheral artery surgery | 10082470 |
| 102 | Peripheral artery thrombosis | 10072564 |
| 103 | Peripheral embolism | 10061340 |
| 104 | Peripheral endarterectomy | 10072560 |
| 105 | Popliteal artery entrapment syndrome | 10071642 |
| 106 | Post procedural myocardial infarction | 10066592 |
| 107 | Postinfarction angina | 10058144 |
| 108 | Precerebral artery occlusion | 10036511 |
| 109 | Precerebral artery thrombosis | 10074717 |
| 110 | Profundaplasty | 10078867 |
| 111 | Pulmonary artery occlusion | 10078201 |
| 112 | Pulmonary artery therapeutic procedure | 10063731 |
| 113 | Pulmonary artery thrombosis | 10037340 |
| 114 | Pulmonary endarterectomy | 10072893 |
| 115 | Pulmonary tumour thrombotic microangiopathy | 10079988 |
| 116 | Renal artery angioplasty | 10057493 |
| 117 | Renal artery occlusion | 10048988 |
| 118 | Renal artery thrombosis | 10038380 |
| 119 | Renal embolism | 10063544 |
| 120 | Retinal artery embolism | 10038826 |
| 121 | Retinal artery occlusion | 10038827 |
| 122 | Retinal artery thrombosis | 10038831 |
| 123 | Silent myocardial infarction | 10049768 |
| 124 | Spinal artery embolism | 10049440 |
| 125 | Spinal artery thrombosis | 10071316 |
| 126 | Splenic artery thrombosis | 10074600 |
| 127 | Splenic embolism | 10068677 |
| 128 | Stress cardiomyopathy | 10066286 |
| 129 | Stroke in evolution | 10059613 |
| 130 | Subclavian artery embolism | 10042332 |
| 131 | Subclavian artery occlusion | 10069695 |
| 132 | Subclavian artery thrombosis | 10042334 |
| 133 | Superior mesenteric artery syndrome | 10054156 |
| 134 | Thromboembolectomy | 10064958 |
| 135 | Thrombotic microangiopathy | 10043645 |
| 136 | Thrombotic thrombocytopenic purpura | 10043648 |
| 137 | Transient ischaemic attack | 10044390 |
| 138 | Truncus coeliacus thrombosis | 10062363 |
| 139 | Vascular pseudoaneurysm thrombosis | 10078269 |
| 140 | Vertebral artery occlusion | 10048965 |
| 141 | Vertebral artery thrombosis | 10057777 |
| 142 | Visual acuity reduced transiently | 10047532 |
| **Venous embolic and thrombotic events** | **Preferred term** | **Code** |
| 1 | Axillary vein thrombosis | 10003880 |
| 2 | Brachiocephalic vein occlusion | 10076837 |
| 3 | Brachiocephalic vein thrombosis | 10063363 |
| 4 | Budd-Chiari syndrome | 10006537 |
| 5 | Catheterisation venous | 10052698 |
| 6 | Cavernous sinus thrombosis | 10007830 |
| 7 | Central venous catheterisation | 10053377 |
| 8 | Cerebral venous sinus thrombosis | 10083037 |
| 9 | Cerebral venous thrombosis | 10008138 |
| 10 | Compression garment application | 10079209 |
| 11 | Deep vein thrombosis | 10051055 |
| 12 | Deep vein thrombosis postoperative | 10066881 |
| 13 | Embolism venous | 10014522 |
| 14 | Hepatic vein embolism | 10078810 |
| 15 | Hepatic vein occlusion | 10058991 |
| 16 | Hepatic vein thrombosis | 10019713 |
| 17 | Homans' sign positive | 10051031 |
| 18 | Iliac vein occlusion | 10058992 |
| 19 | Inferior vena cava syndrome | 10070911 |
| 20 | Inferior vena caval occlusion | 10058987 |
| 21 | Jugular vein embolism | 10081850 |
| 22 | Jugular vein occlusion | 10076835 |
| 23 | Jugular vein thrombosis | 10023237 |
| 24 | Mahler sign | 10075428 |
| 25 | May-Thurner syndrome | 10069727 |
| 26 | Mesenteric vein thrombosis | 10027402 |
| 27 | Mesenteric venous occlusion | 10027403 |
| 28 | Obstetrical pulmonary embolism | 10029925 |
| 29 | Obstructive shock | 10073708 |
| 30 | Ophthalmic vein thrombosis | 10074349 |
| 31 | Ovarian vein thrombosis | 10072059 |
| 32 | Paget-Schroetter syndrome | 10050216 |
| 33 | Pelvic venous thrombosis | 10034272 |
| 34 | Penile vein thrombosis | 10034324 |
| 35 | Peripheral vein occlusion | 10083103 |
| 36 | Peripheral vein thrombus extension | 10082853 |
| 37 | Phlebectomy | 10048874 |
| 38 | Portal vein cavernous transformation | 10073979 |
| 39 | Portal vein embolism | 10082030 |
| 40 | Portal vein occlusion | 10058989 |
| 41 | Portal vein thrombosis | 10036206 |
| 42 | Portosplenomesenteric venous thrombosis | 10077623 |
| 43 | Post procedural pulmonary embolism | 10063909 |
| 44 | Post thrombotic syndrome | 10048591 |
| 45 | Postoperative thrombosis | 10050902 |
| 46 | Postpartum venous thrombosis | 10036300 |
| 47 | Pulmonary embolism | 10037377 |
| 48 | Pulmonary infarction | 10037410 |
| 49 | Pulmonary microemboli | 10037421 |
| 50 | Pulmonary oil microembolism | 10069388 |
| 51 | Pulmonary thrombosis | 10037437 |
| 52 | Pulmonary vein occlusion | 10068690 |
| 53 | Pulmonary veno-occlusive disease | 10037458 |
| 54 | Pulmonary venous thrombosis | 10037459 |
| 55 | Renal vein embolism | 10038547 |
| 56 | Renal vein occlusion | 10056293 |
| 57 | Renal vein thrombosis | 10038548 |
| 58 | Retinal vein occlusion | 10038907 |
| 59 | Retinal vein thrombosis | 10038908 |
| 60 | Septic pulmonary embolism | 10083093 |
| 61 | SI QIII TIII pattern | 10068479 |
| 62 | Splenic vein occlusion | 10068122 |
| 63 | Splenic vein thrombosis | 10041659 |
| 64 | Subclavian vein occlusion | 10079164 |
| 65 | Subclavian vein thrombosis | 10049446 |
| 66 | Superior sagittal sinus thrombosis | 10042567 |
| 67 | Superior vena cava occlusion | 10058988 |
| 68 | Superior vena cava syndrome | 10042569 |
| 69 | Thrombophlebitis | 10043570 |
| 70 | Thrombophlebitis migrans | 10043581 |
| 71 | Thrombophlebitis neonatal | 10043586 |
| 72 | Thrombophlebitis superficial | 10043595 |
| 73 | Thrombosed varicose vein | 10043605 |
| 74 | Thrombosis corpora cavernosa | 10067270 |
| 75 | Transverse sinus thrombosis | 10044457 |
| 76 | Vascular graft | 10067740 |
| 77 | Vena cava embolism | 10047193 |
| 78 | Vena cava filter insertion | 10048932 |
| 79 | Vena cava filter removal | 10074397 |
| 80 | Vena cava thrombosis | 10047195 |
| 81 | Venogram abnormal | 10047209 |
| 82 | Venoocclusive disease | 10062173 |
| 83 | Venoocclusive liver disease | 10047216 |
| 84 | Venous angioplasty | 10077826 |
| 85 | Venous occlusion | 10058990 |
| 86 | Venous operation | 10062175 |
| 87 | Venous recanalisation | 10068605 |
| 88 | Venous repair | 10052964 |
| 89 | Venous stent insertion | 10063389 |
| 90 | Venous thrombosis | 10047249 |
| 91 | Venous thrombosis in pregnancy | 10067030 |
| 92 | Venous thrombosis limb | 10061408 |
| 93 | Venous thrombosis neonatal | 10064602 |
| 94 | Visceral venous thrombosis | 10077829 |
| **Vessel type unspecified and mixed arterial and venous embolic and thrombotic events** | **Preferred term** | **Code** |
| 1 | Administration site thrombosis | 10075968 |
| 2 | Adrenal thrombosis | 10075178 |
| 3 | Angiogram abnormal | 10060956 |
| 4 | Angiogram cerebral abnormal | 10052906 |
| 5 | Angiogram peripheral abnormal | 10057517 |
| 6 | Angioplasty | 10002475 |
| 7 | Antiphospholipid syndrome | 10002817 |
| 8 | Application site thrombosis | 10076026 |
| 9 | Arteriovenous fistula occlusion | 10058562 |
| 10 | Arteriovenous fistula thrombosis | 10003192 |
| 11 | Arteriovenous graft thrombosis | 10053182 |
| 12 | Artificial blood vessel occlusion | 10078895 |
| 13 | Atrial thrombosis | 10048632 |
| 14 | Basal ganglia stroke | 10071043 |
| 15 | Bone infarction | 10049824 |
| 16 | Brain stem embolism | 10074422 |
| 17 | Brain stem infarction | 10006147 |
| 18 | Brain stem stroke | 10068644 |
| 19 | Brain stem thrombosis | 10062573 |
| 20 | Cardiac ventricular thrombosis | 10053994 |
| 21 | Catheter site thrombosis | 10079523 |
| 22 | Cerebellar embolism | 10067167 |
| 23 | Cerebellar infarction | 10008034 |
| 24 | Cerebral congestion | 10076929 |
| 25 | Cerebral infarction | 10008118 |
| 26 | Cerebral infarction foetal | 10008119 |
| 27 | Cerebral ischaemia | 10008120 |
| 28 | Cerebral microembolism | 10078311 |
| 29 | Cerebral microinfarction | 10083668 |
| 30 | Cerebral septic infarct | 10070671 |
| 31 | Cerebral thrombosis | 10008132 |
| 32 | Cerebral vascular occlusion | 10076895 |
| 33 | Cerebrospinal thrombotic tamponade | 10052173 |
| 34 | Cerebrovascular accident | 10008190 |
| 35 | Cerebrovascular accident prophylaxis | 10049165 |
| 36 | Cerebrovascular disorder | 10008196 |
| 37 | Cerebrovascular operation | 10051902 |
| 38 | Choroidal infarction | 10057403 |
| 39 | Collateral circulation | 10069729 |
| 40 | Coronary angioplasty | 10050329 |
| 41 | Coronary artery thrombosis | 10011091 |
| 42 | Coronary bypass thrombosis | 10059025 |
| 43 | Device embolisation | 10074896 |
| 44 | Device occlusion | 10064685 |
| 45 | Device related thrombosis | 10077455 |
| 46 | Diplegia | 10013033 |
| 47 | Directional Doppler flow tests abnormal | 10013048 |
| 48 | Disseminated intravascular coagulation | 10013442 |
| 49 | Disseminated intravascular coagulation in newborn | 10013443 |
| 50 | Embolic cerebellar infarction | 10084072 |
| 51 | Embolic cerebral infarction | 10060839 |
| 52 | Embolic pneumonia | 10065680 |
| 53 | Embolic stroke | 10014498 |
| 54 | Embolism | 10061169 |
| 55 | Eye infarction | 10083006 |
| 56 | Fluorescence angiogram abnormal | 10083087 |
| 57 | Foetal cerebrovascular disorder | 10053601 |
| 58 | Graft thrombosis | 10051269 |
| 59 | Haemorrhagic adrenal infarction | 10079902 |
| 60 | Haemorrhagic cerebral infarction | 10019005 |
| 61 | Haemorrhagic infarction | 10019013 |
| 62 | Haemorrhagic stroke | 10019016 |
| 63 | Haemorrhagic transformation stroke | 10055677 |
| 64 | Haemorrhoids thrombosed | 10019023 |
| 65 | Hemiparesis | 10019465 |
| 66 | Hemiplegia | 10019468 |
| 67 | Heparin-induced thrombocytopenia | 10062506 |
| 68 | Hepatic infarction | 10019680 |
| 69 | Hepatic vascular thrombosis | 10074494 |
| 70 | Implant site thrombosis | 10063868 |
| 71 | Incision site vessel occlusion | 10076839 |
| 72 | Infarction | 10061216 |
| 73 | Infusion site thrombosis | 10065489 |
| 74 | Injection site thrombosis | 10022104 |
| 75 | Inner ear infarction | 10070754 |
| 76 | Instillation site thrombosis | 10073625 |
| 77 | Intestinal infarction | 10022657 |
| 78 | Intracardiac mass | 10066087 |
| 79 | Intracardiac thrombus | 10048620 |
| 80 | Lambl's excrescences | 10083691 |
| 81 | Medical device site thrombosis | 10076145 |
| 82 | Mesenteric arterial occlusion | 10027394 |
| 83 | Mesenteric vascular insufficiency | 10027401 |
| 84 | Mesenteric vascular occlusion | 10074583 |
| 85 | Mesenteric venous occlusion | 10027403 |
| 86 | Microembolism | 10073734 |
| 87 | Monoparesis | 10027925 |
| 88 | Monoplegia | 10027926 |
| 89 | Optic nerve infarction | 10030936 |
| 90 | Pancreatic infarction | 10068239 |
| 91 | Paradoxical embolism | 10066059 |
| 92 | Paraneoplastic thrombosis | 10079251 |
| 93 | Paraparesis | 10033885 |
| 94 | Paraplegia | 10033892 |
| 95 | Paresis | 10033985 |
| 96 | Peripheral revascularisation | 10053351 |
| 97 | Pituitary infarction | 10035092 |
| 98 | Placental infarction | 10064620 |
| 99 | Pneumatic compression therapy | 10059829 |
| 100 | Portal shunt | 10036204 |
| 101 | Portal shunt procedure | 10077479 |
| 102 | Post procedural stroke | 10066591 |
| 103 | Postpartum thrombosis | 10077022 |
| 104 | Prosthetic cardiac valve thrombosis | 10063176 |
| 105 | Prosthetic vessel implantation | 10068628 |
| 106 | Quadriparesis | 10049680 |
| 107 | Quadriplegia | 10037714 |
| 108 | Renal artery angioplasty | 10057493 |
| 109 | Renal infarct | 10038470 |
| 110 | Renal vascular thrombosis | 10072226 |
| 111 | Retinal infarction | 10051742 |
| 112 | Retinal vascular thrombosis | 10062108 |
| 113 | Revascularisation procedure | 10084091 |
| 114 | Shunt occlusion | 10040621 |
| 115 | Shunt thrombosis | 10059054 |
| 116 | Silent myocardial infarction | 10049768 |
| 117 | Spinal cord infarction | 10058571 |
| 118 | Spinal stroke | 10082031 |
| 119 | Splenic infarction | 10041648 |
| 120 | Splenic thrombosis | 10074601 |
| 121 | Stoma site thrombosis | 10074515 |
| 122 | Stroke in evolution | 10059613 |
| 123 | Surgical vascular shunt | 10058408 |
| 124 | Testicular infarction | 10043337 |
| 125 | Thalamic infarction | 10064961 |
| 126 | Thrombectomy | 10043530 |
| 127 | Thromboangiitis obliterans | 10043540 |
| 128 | Thrombolysis | 10043568 |
| 129 | Thrombosis | 10043607 |
| 130 | Thrombosis in device | 10062546 |
| 131 | Thrombosis mesenteric vessel | 10043626 |
| 132 | Thrombosis prophylaxis | 10043634 |
| 133 | Thrombotic cerebral infarction | 10067347 |
| 134 | Thrombotic stroke | 10043647 |
| 135 | Thyroid infarction | 10043742 |
| 136 | Tumour embolism | 10045168 |
| 137 | Tumour thrombectomy | 10081994 |
| 138 | Tumour thrombosis | 10068067 |
| 139 | Ultrasonic angiogram abnormal | 10061604 |
| 140 | Ultrasound Doppler abnormal | 10045413 |
| 141 | Umbilical cord occlusion | 10076714 |
| 142 | Umbilical cord thrombosis | 10071652 |
| 143 | Vaccination site thrombosis | 10076190 |
| 144 | Vascular access site thrombosis | 10078675 |
| 145 | Vascular device occlusion | 10080803 |
| 146 | Vascular graft | 10067740 |
| 147 | Vascular graft occlusion | 10049060 |
| 148 | Vascular graft thrombosis | 10069922 |
| 149 | Vascular operation | 10049071 |
| 150 | Vascular stent insertion | 10063382 |
| 151 | Vascular stent occlusion | 10077143 |
| 152 | Vascular stent thrombosis | 10063934 |
| 153 | Vasodilation procedure | 10058794 |
| 154 | Vessel puncture site occlusion | 10076838 |
| 155 | Vessel puncture site thrombosis | 10070649 |
| 156 | Visual midline shift syndrome | 10066856 |
