# Supplementary material for: Serious adverse reaction associated with the COVID-19 vaccines of BNT162b2, Ad26.COV2.S, and mRNA-1273: Gaining insight through the VAERS
Source: Front Pharmacol. 2022 Nov 7;13:921760. doi: 10.3389/fphar.2022.921760 (PMC9676979; doi:10.3389/fphar.2022.921760)
Supplement: Supplementary file 13 [file Table5.DOCX]

Supplementary Table 4 The preferred term of cardiac arrhythmias used in this study.

|  | **Arrhythmia related investigations, signs and symptoms (SMQ)** | |
| --- | --- | --- |
|  | **Preferred term** | **Code** |
| 1 | Chronotropic incompetence | 10068627 |
| 2 | Electrocardiogram repolarisation abnormality | 10052464 |
| 3 | Electrocardiogram RR interval prolonged | 10067652 |
| 4 | Electrocardiogram U wave inversion | 10062314 |
| 5 | Electrocardiogram U wave present | 10057913 |
| 6 | Electrocardiogram U-wave abnormality | 10055032 |
| 7 | Sudden cardiac death | 10049418 |
| 8 | Bezold-Jarisch reflex | 10076999 |
| 9 | Bradycardia | 10006093 |
| 10 | Cardiac arrest | 10007515 |
| 11 | Cardiac death | 10049993 |
| 12 | Cardiac telemetry abnormal | 10053450 |
| 13 | Cardio-respiratory arrest | 10007617 |
| 14 | Central bradycardia | 10078310 |
| 15 | Electrocardiogram abnormal | 10014363 |
| 16 | Electrocardiogram ambulatory abnormal | 10014369 |
| 17 | Electrocardiogram change | 10061116 |
| 18 | Heart rate abnormal | 10019300 |
| 19 | Heart rate decreased | 10019301 |
| 20 | Heart rate increased | 10019303 |
| 21 | Loss of consciousness | 10024855 |
| 22 | Palpitations | 10033557 |
| 23 | Rebound tachycardia | 10067207 |
| 24 | Respiratory sinus arrhythmia magnitude abnormal | 10079117 |
| 25 | Respiratory sinus arrhythmia magnitude decreased | 10079116 |
| 26 | Respiratory sinus arrhythmia magnitude increased | 10079115 |
| 27 | Sudden death | 10042434 |
| 28 | Syncope | 10042772 |
| 29 | Tachycardia | 10043071 |
| 30 | Tachycardia paroxysmal | 10043079 |
|  | **Bradyarrhythmia terms, nonspecific (SMQ)** | |
| 1 | Bradyarrhythmia | 10049765 |
| 2 | Ventricular asystole | 10047284 |
|  | **Conduction defects (SMQ)** | |
| 1 | Accessory cardiac pathway | 10067618 |
| 2 | Adams-Stokes syndrome | 10001115 |
| 3 | Agonal rhythm | 10054015 |
| 4 | Atrial conduction time prolongation | 10064191 |
| 5 | Atrioventricular block | 10003671 |
| 6 | Atrioventricular block complete | 10003673 |
| 7 | Atrioventricular block first degree | 10003674 |
| 8 | Atrioventricular block second degree | 10003677 |
| 9 | Atrioventricular conduction time shortened | 10068180 |
| 10 | Atrioventricular dissociation | 10069571 |
| 11 | Atrioventricular node dysfunction | 10084085 |
| 12 | Bifascicular block | 10057393 |
| 13 | BRASH syndrome | 10084073 |
| 14 | Brugada syndrome | 10059027 |
| 15 | Bundle branch block | 10006578 |
| 16 | Bundle branch block bilateral | 10006579 |
| 17 | Bundle branch block left | 10006580 |
| 18 | Bundle branch block right | 10006582 |
| 19 | Conduction disorder | 10010276 |
| 20 | Defect conduction intraventricular | 10012118 |
| 21 | Electrocardiogram delta waves abnormal | 10014372 |
| 22 | Electrocardiogram PR prolongation | 10053657 |
| 23 | Electrocardiogram PR shortened | 10014374 |
| 24 | Electrocardiogram QRS complex prolonged | 10014380 |
| 25 | Electrocardiogram QT prolonged | 10014387 |
| 26 | Electrocardiogram repolarisation abnormality | 10052464 |
| 27 | Lenegre's disease | 10071710 |
| 28 | Long QT syndrome | 10024803 |
| 29 | Paroxysmal atrioventricular block | 10077503 |
| 30 | Sinoatrial block | 10040736 |
| 31 | Trifascicular block | 10044644 |
| 32 | Ventricular dyssynchrony | 10071186 |
| 33 | Wolff-Parkinson-White syndrome | 10048015 |
|  | **Disorders of sinus node function (SMQ)** |  |
| 1 | Nodal arrhythmia | 10029458 |
| 2 | Nodal rhythm | 10029470 |
| 3 | Sinus arrest | 10040738 |
| 4 | Sinus arrhythmia | 10040739 |
| 5 | Sinus bradycardia | 10040741 |
| 6 | Sinus node dysfunction | 10075889 |
| 7 | Wandering pacemaker | 10047818 |
|  | **Cardiac arrhythmia terms, nonspecific (SMQ)** |  |
| 1 | Arrhythmia | 10003119 |
| 2 | Heart alternation | 10058155 |
| 3 | Heart rate irregular | 10019304 |
| 4 | Holiday heart syndrome | 10083709 |
| 5 | Pacemaker generated arrhythmia | 10053486 |
| 6 | Pacemaker syndrome | 10051994 |
| 7 | Paroxysmal arrhythmia | 10050106 |
| 8 | Pulseless electrical activity | 10058151 |
| 9 | Reperfusion arrhythmia | 10058156 |
| 10 | Withdrawal arrhythmia | 10047997 |
|  | **Supraventricular tachyarrhythmias (SMQ)** | |
| 1 | Arrhythmia supraventricular | 10003130 |
| 2 | Atrial fibrillation | 10003658 |
| 3 | Atrial flutter | 10003662 |
| 4 | Atrial parasystole | 10071666 |
| 5 | Atrial tachycardia | 10003668 |
| 6 | Congenital supraventricular tachycardia | 10082343 |
| 7 | Frederick's syndrome | 10082089 |
| 8 | Junctional ectopic tachycardia | 10074640 |
| 9 | Sinus tachycardia | 10040752 |
| 10 | Supraventricular extrasystoles | 10042602 |
| 11 | Supraventricular tachyarrhythmia | 10065342 |
| 12 | Supraventricular tachycardia | 10042604 |
| 13 | ECG P wave inverted | 10057526 |
| 14 | Electrocardiogram P wave abnormal | 10050384 |
| 15 | Retrograde p-waves | 10071187 |
|  | **Tachyarrhythmia terms, nonspecific (SMQ)** | |
| 1 | Anomalous atrioventricular excitation | 10002611 |
| 2 | Cardiac fibrillation | 10061592 |
| 3 | Cardiac flutter | 10052840 |
| 4 | Extrasystoles | 10015856 |
| 5 | Tachyarrhythmia | 10049447 |
|  | **Ventricular tachyarrhythmias (SMQ)** | |
| 1 | Accelerated idioventricular rhythm | 10049003 |
| 2 | Cardiac fibrillation | 10061592 |
| 3 | Parasystole | 10033929 |
| 4 | Rhythm idioventricular | 10039111 |
| 5 | Torsade de pointes | 10044066 |
| 6 | Ventricular arrhythmia | 10047281 |
| 7 | Ventricular extrasystoles | 10047289 |
| 8 | Ventricular fibrillation | 10047290 |
| 9 | Ventricular flutter | 10047294 |
| 10 | Ventricular parasystole | 10058184 |
| 11 | Ventricular pre-excitation | 10049761 |
| 12 | Ventricular tachyarrhythmia | 10065341 |
| 13 | Ventricular tachycardia | 10047302 |
