# Supplementary material for: Serious adverse reaction associated with the COVID-19 vaccines of BNT162b2, Ad26.COV2.S, and mRNA-1273: Gaining insight through the VAERS
Source: Front Pharmacol. 2022 Nov 7;13:921760. doi: 10.3389/fphar.2022.921760 (PMC9676979; doi:10.3389/fphar.2022.921760)
Supplement: Supplementary file 14 [file Table3.DOCX]

Supplementary Table 2 The preferred term of haemorrhage used in this study.

| **Haemorrhage** **events** | **Preferred term** | **Code** |
| --- | --- | --- |
| 1 | Abdominal wall haematoma | 10067383 |
| 2 | Abdominal wall haemorrhage | 10067788 |
| 3 | Abnormal withdrawal bleeding | 10069195 |
| 4 | Achenbach syndrome | 10079562 |
| 5 | Acute haemorrhagic leukoencephalitis | 10058994 |
| 6 | Acute haemorrhagic ulcerative colitis | 10075634 |
| 7 | Administration site bruise | 10075094 |
| 8 | Administration site haematoma | 10075100 |
| 9 | Administration site haemorrhage | 10075101 |
| 10 | Adrenal haematoma | 10059194 |
| 11 | Adrenal haemorrhage | 10001361 |
| 12 | Anal fissure haemorrhage | 10079765 |
| 13 | Anal haemorrhage | 10049555 |
| 14 | Anal ulcer haemorrhage | 10063896 |
| 15 | Anastomotic haemorrhage | 10056346 |
| 16 | Anastomotic ulcer haemorrhage | 10002244 |
| 17 | Aneurysm ruptured | 10048380 |
| 18 | Angina bullosa haemorrhagica | 10064223 |
| 19 | Anorectal varices haemorrhage | 10068925 |
| 20 | Anticoagulant-related nephropathy | 10083346 |
| 21 | Aortic aneurysm rupture | 10002886 |
| 22 | Aortic dissection rupture | 10068119 |
| 23 | Aortic intramural haematoma | 10067975 |
| 24 | Aortic perforation | 10075729 |
| 25 | Aortic rupture | 10060874 |
| 26 | Aponeurosis contusion | 10075330 |
| 27 | Application site bruise | 10050114 |
| 28 | Application site haematoma | 10068317 |
| 29 | Application site haemorrhage | 10072694 |
| 30 | Application site purpura | 10050182 |
| 31 | Arterial haemorrhage | 10060964 |
| 32 | Arterial intramural haematoma | 10074971 |
| 33 | Arterial perforation | 10075732 |
| 34 | Arterial rupture | 10003173 |
| 35 | Arteriovenous fistula site haematoma | 10055150 |
| 36 | Arteriovenous fistula site haemorrhage | 10055123 |
| 37 | Arteriovenous graft site haematoma | 10055152 |
| 38 | Arteriovenous graft site haemorrhage | 10055126 |
| 39 | Astringent therapy | 10067372 |
| 40 | Atrial rupture | 10048761 |
| 41 | Auricular haematoma | 10003797 |
| 42 | Basal ganglia haematoma | 10077031 |
| 43 | Basal ganglia haemorrhage | 10067057 |
| 44 | Basilar artery perforation | 10075736 |
| 45 | Bladder tamponade | 10062656 |
| 46 | Bleeding varicose vein | 10005144 |
| 47 | Blood blister | 10005372 |
| 48 | Blood loss anaemia | 10082297 |
| 49 | Blood urine | 10005863 |
| 50 | Blood urine present | 10018870 |
| 51 | Bloody discharge | 10057687 |
| 52 | Bloody peritoneal effluent | 10067442 |
| 53 | Bone contusion | 10066251 |
| 54 | Bone marrow haemorrhage | 10073581 |
| 55 | Brain contusion | 10052346 |
| 56 | Brain stem haematoma | 10073230 |
| 57 | Brain stem haemorrhage | 10006145 |
| 58 | Brain stem microhaemorrhage | 10071205 |
| 59 | Breast haematoma | 10064753 |
| 60 | Breast haemorrhage | 10006254 |
| 61 | Broad ligament haematoma | 10006375 |
| 62 | Bronchial haemorrhage | 10065739 |
| 63 | Bronchial varices haemorrhage | 10079163 |
| 64 | Bullous haemorrhagic dermatosis | 10083809 |
| 65 | Bursal haematoma | 10077818 |
| 66 | Cardiac contusion | 10073356 |
| 67 | Carotid aneurysm rupture | 10051328 |
| 68 | Carotid artery perforation | 10075728 |
| 69 | Catheter site bruise | 10063587 |
| 70 | Catheter site haematoma | 10055662 |
| 71 | Catheter site haemorrhage | 10051099 |
| 72 | Central nervous system haemorrhage | 10072043 |
| 73 | Cephalhaematoma | 10008014 |
| 74 | Cerebellar haematoma | 10061038 |
| 75 | Cerebellar haemorrhage | 10008030 |
| 76 | Cerebellar microhaemorrhage | 10071206 |
| 77 | Cerebral aneurysm perforation | 10075394 |
| 78 | Cerebral aneurysm ruptured syphilitic | 10008076 |
| 79 | Cerebral arteriovenous malformation haemorrhagic | 10008086 |
| 80 | Cerebral artery perforation | 10075734 |
| 81 | Cerebral cyst haemorrhage | 10082099 |
| 82 | Cerebral haematoma | 10053942 |
| 83 | Cerebral haemorrhage | 10008111 |
| 84 | Cerebral haemorrhage foetal | 10050157 |
| 85 | Cerebral haemorrhage neonatal | 10008112 |
| 86 | Cerebral microhaemorrhage | 10067277 |
| 87 | Cervix haematoma uterine | 10050020 |
| 88 | Cervix haemorrhage uterine | 10050022 |
| 89 | Chest wall haematoma | 10076597 |
| 90 | Choroidal haematoma | 10068642 |
| 91 | Choroidal haemorrhage | 10008786 |
| 92 | Chronic gastrointestinal bleeding | 10050399 |
| 93 | Chronic pigmented purpura | 10072726 |
| 94 | Ciliary body haemorrhage | 10057417 |
| 95 | Coital bleeding | 10065019 |
| 96 | Colonic haematoma | 10009996 |
| 97 | Conjunctival haemorrhage | 10010719 |
| 98 | Contusion | 10050584 |
| 99 | Corneal bleeding | 10051558 |
| 100 | Cullen's sign | 10059029 |
| 101 | Cystitis haemorrhagic | 10011793 |
| 102 | Deep dissecting haematoma | 10074718 |
| 103 | Diarrhoea haemorrhagic | 10012741 |
| 104 | Disseminated intravascular coagulation | 10013442 |
| 105 | Diverticulitis intestinal haemorrhagic | 10013541 |
| 106 | Diverticulum intestinal haemorrhagic | 10013560 |
| 107 | Duodenal ulcer haemorrhage | 10013839 |
| 108 | Duodenitis haemorrhagic | 10013865 |
| 109 | Dysfunctional uterine bleeding | 10013908 |
| 110 | Ear haemorrhage | 10014009 |
| 111 | Ecchymosis | 10014080 |
| 112 | Encephalitis haemorrhagic | 10014589 |
| 113 | Enterocolitis haemorrhagic | 10014896 |
| 114 | Epidural haemorrhage | 10073681 |
| 115 | Epistaxis | 10015090 |
| 116 | Exsanguination | 10015719 |
| 117 | Extra-axial haemorrhage | 10078254 |
| 118 | Extradural haematoma | 10015769 |
| 119 | Extradural haematoma evacuation | 10082797 |
| 120 | Extravasation blood | 10015867 |
| 121 | Eye contusion | 10073354 |
| 122 | Eye haematoma | 10079891 |
| 123 | Eye haemorrhage | 10015926 |
| 124 | Eyelid bleeding | 10053196 |
| 125 | Eyelid contusion | 10075018 |
| 126 | Eyelid haematoma | 10064976 |
| 127 | Femoral artery perforation | 10075739 |
| 128 | Femoral vein perforation | 10075745 |
| 129 | Foetal-maternal haemorrhage | 10016871 |
| 130 | Fothergill sign positive | 10081749 |
| 131 | Gastric haemorrhage | 10017788 |
| 132 | Gastric ulcer haemorrhage | 10017826 |
| 133 | Gastric ulcer haemorrhage, obstructive | 10017829 |
| 134 | Gastric ulcer perforation | 10017835 |
| 135 | Gastric varices haemorrhage | 10057572 |
| 136 | Gastritis alcoholic haemorrhagic | 10017857 |
| 137 | Gastritis haemorrhagic | 10017866 |
| 138 | Gastroduodenal haemorrhage | 10053768 |
| 139 | Gastrointestinal haemorrhage | 10017955 |
| 140 | Gastrointestinal polyp haemorrhage | 10074437 |
| 141 | Gastrointestinal ulcer haemorrhage | 10056743 |
| 142 | Gastrointestinal vascular malformation haemorrhagic | 10080561 |
| 143 | Genital contusion | 10073355 |
| 144 | Genital haemorrhage | 10061178 |
| 145 | Gingival bleeding | 10018276 |
| 146 | Graft haemorrhage | 10063577 |
| 147 | Grey Turner's sign | 10075426 |
| 148 | Haemangioma rupture | 10084040 |
| 149 | Haemarthrosis | 10018829 |
| 150 | Haematemesis | 10018830 |
| 151 | Haematochezia | 10018836 |
| 152 | Haematocoele | 10018833 |
| 153 | Haematoma | 10018852 |
| 154 | Haematoma evacuation | 10060733 |
| 155 | Haematoma infection | 10051564 |
| 156 | Haematoma muscle | 10055890 |
| 157 | Haematosalpinx | 10050468 |
| 158 | Haematospermia | 10018866 |
| 159 | Haematotympanum | 10063013 |
| 160 | Haematuria | 10018867 |
| 161 | Haematuria traumatic | 10018871 |
| 162 | Haemobilia | 10058947 |
| 163 | Haemoperitoneum | 10018935 |
| 164 | Haemophilic arthropathy | 10065057 |
| 165 | Haemophilic pseudotumour | 10073770 |
| 166 | Haemoptysis | 10018964 |
| 167 | Haemorrhage | 10055798 |
| 168 | Haemorrhage coronary artery | 10055803 |
| 169 | Haemorrhage foetal | 10061191 |
| 170 | Haemorrhage in pregnancy | 10018981 |
| 171 | Haemorrhage intracranial | 10018985 |
| 172 | Haemorrhage neonatal | 10061993 |
| 173 | Haemorrhage subcutaneous | 10018999 |
| 174 | Haemorrhage subepidermal | 10019001 |
| 175 | Haemorrhage urinary tract | 10055847 |
| 176 | Haemorrhagic adrenal infarction | 10079902 |
| 177 | Haemorrhagic arteriovenous malformation | 10064595 |
| 178 | Haemorrhagic ascites | 10059766 |
| 179 | Haemorrhagic breast cyst | 10077443 |
| 180 | Haemorrhagic cerebral infarction | 10019005 |
| 181 | Haemorrhagic cyst | 10059189 |
| 182 | Haemorrhagic diathesis | 10062713 |
| 183 | Haemorrhagic disease of newborn | 10019008 |
| 184 | Haemorrhagic disorder | 10019009 |
| 185 | Haemorrhagic erosive gastritis | 10067786 |
| 186 | Haemorrhagic hepatic cyst | 10067796 |
| 187 | Haemorrhagic infarction | 10019013 |
| 188 | Haemorrhagic necrotic pancreatitis | 10076058 |
| 189 | Haemorrhagic ovarian cyst | 10060781 |
| 190 | Haemorrhagic stroke | 10019016 |
| 191 | Haemorrhagic thyroid cyst | 10072256 |
| 192 | Haemorrhagic transformation stroke | 10055677 |
| 193 | Haemorrhagic tumour necrosis | 10054096 |
| 194 | Haemorrhagic urticaria | 10059499 |
| 195 | Haemorrhagic vasculitis | 10071252 |
| 196 | Haemorrhoidal haemorrhage | 10054787 |
| 197 | Haemostasis | 10067439 |
| 198 | Haemothorax | 10019027 |
| 199 | Henoch-Schonlein purpura | 10019617 |
| 200 | Hepatic haemangioma rupture | 10054885 |
| 201 | Hepatic haematoma | 10019676 |
| 202 | Hepatic haemorrhage | 10019677 |
| 203 | Hereditary haemorrhagic telangiectasia | 10019883 |
| 204 | Hyperfibrinolysis | 10074737 |
| 205 | Hyphaema | 10020923 |
| 206 | Iliac artery perforation | 10075731 |
| 207 | Iliac artery rupture | 10072789 |
| 208 | Iliac vein perforation | 10075744 |
| 209 | Immune thrombocytopenia | 10083842 |
| 210 | Implant site bruising | 10063850 |
| 211 | Implant site haematoma | 10063780 |
| 212 | Implant site haemorrhage | 10053995 |
| 213 | Incision site haematoma | 10059241 |
| 214 | Incision site haemorrhage | 10051100 |
| 215 | Increased tendency to bruise | 10021688 |
| 216 | Induced abortion haemorrhage | 10052844 |
| 217 | Inferior vena cava perforation | 10075742 |
| 218 | Infusion site bruising | 10059203 |
| 219 | Infusion site haematoma | 10065463 |
| 220 | Infusion site haemorrhage | 10065464 |
| 221 | Injection site bruising | 10022052 |
| 222 | Injection site haematoma | 10022066 |
| 223 | Injection site haemorrhage | 10022067 |
| 224 | Instillation site bruise | 10073630 |
| 225 | Instillation site haematoma | 10073609 |
| 226 | Instillation site haemorrhage | 10073610 |
| 227 | Internal haemorrhage | 10075192 |
| 228 | Intestinal haematoma | 10069829 |
| 229 | Intestinal haemorrhage | 10059175 |
| 230 | Intestinal varices haemorrhage | 10078058 |
| 231 | Intra-abdominal haematoma | 10056457 |
| 232 | Intra-abdominal haemorrhage | 10061249 |
| 233 | Intracerebral haematoma evacuation | 10062025 |
| 234 | Intracranial haematoma | 10059491 |
| 235 | Intracranial tumour haemorrhage | 10022775 |
| 236 | Intraocular haematoma | 10071934 |
| 237 | Intrapartum haemorrhage | 10067703 |
| 238 | Intraventricular haemorrhage | 10022840 |
| 239 | Intraventricular haemorrhage neonatal | 10022841 |
| 240 | Iris haemorrhage | 10057418 |
| 241 | Joint microhaemorrhage | 10077666 |
| 242 | Kidney contusion | 10023413 |
| 243 | Lacrimal haemorrhage | 10069930 |
| 244 | Large intestinal haemorrhage | 10052534 |
| 245 | Large intestinal ulcer haemorrhage | 10061262 |
| 246 | Laryngeal haematoma | 10070885 |
| 247 | Laryngeal haemorrhage | 10065740 |
| 248 | Lip haematoma | 10066304 |
| 249 | Lip haemorrhage | 10049297 |
| 250 | Liver contusion | 10067266 |
| 251 | Lower gastrointestinal haemorrhage | 10050953 |
| 252 | Lower limb artery perforation | 10075730 |
| 253 | Lymph node haemorrhage | 10074270 |
| 254 | Mallory-Weiss syndrome | 10026712 |
| 255 | Mediastinal haematoma | 10049941 |
| 256 | Mediastinal haemorrhage | 10056343 |
| 257 | Medical device site bruise | 10075570 |
| 258 | Medical device site haematoma | 10075577 |
| 259 | Medical device site haemorrhage | 10075578 |
| 260 | Melaena | 10027141 |
| 261 | Melaena neonatal | 10049777 |
| 262 | Meningorrhagia | 10052593 |
| 263 | Menometrorrhagia | 10027295 |
| 264 | Menorrhagia | 10027313 |
| 265 | Mesenteric haematoma | 10071557 |
| 266 | Mesenteric haemorrhage | 10060717 |
| 267 | Metrorrhagia | 10027514 |
| 268 | Mouth haemorrhage | 10028024 |
| 269 | Mucocutaneous haemorrhage | 10076048 |
| 270 | Mucosal haemorrhage | 10061298 |
| 271 | Muscle contusion | 10070757 |
| 272 | Muscle haemorrhage | 10028309 |
| 273 | Myocardial haemorrhage | 10048849 |
| 274 | Myocardial rupture | 10028604 |
| 275 | Naevus haemorrhage | 10062955 |
| 276 | Nail bed bleeding | 10048891 |
| 277 | Nasal septum haematoma | 10075027 |
| 278 | Neonatal gastrointestinal haemorrhage | 10074159 |
| 279 | Nephritis haemorrhagic | 10029132 |
| 280 | Nipple exudate bloody | 10029418 |
| 281 | Occult blood positive | 10061880 |
| 282 | Ocular retrobulbar haemorrhage | 10057571 |
| 283 | Oesophageal haemorrhage | 10030172 |
| 284 | Oesophageal intramural haematoma | 10077486 |
| 285 | Oesophageal ulcer haemorrhage | 10030202 |
| 286 | Oesophageal varices haemorrhage | 10030210 |
| 287 | Oesophagitis haemorrhagic | 10030219 |
| 288 | Optic disc haemorrhage | 10030919 |
| 289 | Optic nerve sheath haemorrhage | 10030941 |
| 290 | Oral blood blister | 10076590 |
| 291 | Oral contusion | 10078170 |
| 292 | Oral mucosa haematoma | 10074779 |
| 293 | Oral purpura | 10083533 |
| 294 | Orbital haematoma | 10083565 |
| 295 | Orbital haemorrhage | 10031045 |
| 296 | Osteorrhagia | 10051937 |
| 297 | Ovarian haematoma | 10033263 |
| 298 | Ovarian haemorrhage | 10065741 |
| 299 | Palpable purpura | 10056872 |
| 300 | Pancreatic haemorrhage | 10033625 |
| 301 | Pancreatic pseudocyst haemorrhage | 10083813 |
| 302 | Pancreatitis haemorrhagic | 10033650 |
| 303 | Papillary muscle haemorrhage | 10059164 |
| 304 | Paranasal sinus haematoma | 10069702 |
| 305 | Paranasal sinus haemorrhage | 10080108 |
| 306 | Parathyroid haemorrhage | 10059051 |
| 307 | Parotid gland haemorrhage | 10051166 |
| 308 | Pelvic haematoma | 10054974 |
| 309 | Pelvic haematoma obstetric | 10034248 |
| 310 | Pelvic haemorrhage | 10063678 |
| 311 | Penile contusion | 10073352 |
| 312 | Penile haematoma | 10070656 |
| 313 | Penile haemorrhage | 10034305 |
| 314 | Peptic ulcer haemorrhage | 10034344 |
| 315 | Pericardial haemorrhage | 10034476 |
| 316 | Perineal haematoma | 10034520 |
| 317 | Periorbital haematoma | 10034544 |
| 318 | Periorbital haemorrhage | 10071697 |
| 319 | Periosteal haematoma | 10077341 |
| 320 | Peripartum haemorrhage | 10072693 |
| 321 | Peripheral artery aneurysm rupture | 10079908 |
| 322 | Peripheral artery haematoma | 10081077 |
| 323 | Peritoneal haematoma | 10058095 |
| 324 | Periventricular haemorrhage neonatal | 10076706 |
| 325 | Petechiae | 10034754 |
| 326 | Pharyngeal contusion | 10083176 |
| 327 | Pharyngeal haematoma | 10068121 |
| 328 | Pharyngeal haemorrhage | 10034827 |
| 329 | Pituitary apoplexy | 10056447 |
| 330 | Pituitary haemorrhage | 10049760 |
| 331 | Placenta praevia haemorrhage | 10035121 |
| 332 | Polymenorrhagia | 10064050 |
| 333 | Post abortion haemorrhage | 10036246 |
| 334 | Post procedural contusion | 10073353 |
| 335 | Post procedural haematoma | 10063188 |
| 336 | Post procedural haematuria | 10066225 |
| 337 | Post procedural haemorrhage | 10051077 |
| 338 | Post transfusion purpura | 10072265 |
| 339 | Postmenopausal haemorrhage | 10055870 |
| 340 | Postpartum haemorrhage | 10036417 |
| 341 | Post-traumatic punctate intraepidermal haemorrhage | 10071639 |
| 342 | Premature separation of placenta | 10036608 |
| 343 | Procedural haemorrhage | 10071229 |
| 344 | Proctitis haemorrhagic | 10036778 |
| 345 | Prostatic haemorrhage | 10036960 |
| 346 | Pulmonary alveolar haemorrhage | 10037313 |
| 347 | Pulmonary contusion | 10037370 |
| 348 | Pulmonary haematoma | 10054991 |
| 349 | Pulmonary haemorrhage | 10037394 |
| 350 | Pulmonary haemorrhage neonatal | 10082194 |
| 351 | Puncture site bruise | 10082035 |
| 352 | Puncture site haematoma | 10081957 |
| 353 | Puncture site haemorrhage | 10051101 |
| 354 | Purpura | 10037549 |
| 355 | Purpura fulminans | 10037556 |
| 356 | Purpura neonatal | 10037557 |
| 357 | Purpura non-thrombocytopenic | 10057739 |
| 358 | Purpura senile | 10037560 |
| 359 | Putamen haemorrhage | 10058940 |
| 360 | Radiation associated haemorrhage | 10072281 |
| 361 | Rectal haemorrhage | 10038063 |
| 362 | Rectal ulcer haemorrhage | 10038081 |
| 363 | Renal artery perforation | 10075737 |
| 364 | Renal cyst haemorrhage | 10059846 |
| 365 | Renal haematoma | 10038459 |
| 366 | Renal haemorrhage | 10038460 |
| 367 | Respiratory tract haemorrhage | 10038727 |
| 368 | Respiratory tract haemorrhage neonatal | 10038728 |
| 369 | Retinal aneurysm rupture | 10079121 |
| 370 | Retinal haemorrhage | 10038867 |
| 371 | Retinopathy haemorrhagic | 10051447 |
| 372 | Retroperitoneal haematoma | 10058360 |
| 373 | Retroperitoneal haemorrhage | 10038980 |
| 374 | Retroplacental haematoma | 10054798 |
| 375 | Ruptured cerebral aneurysm | 10039330 |
| 376 | Scleral haemorrhage | 10050508 |
| 377 | Scrotal haematocoele | 10061517 |
| 378 | Scrotal haematoma | 10039749 |
| 379 | Scrotal haemorrhage | 10061361 |
| 380 | Shock haemorrhagic | 10049771 |
| 381 | Skin haemorrhage | 10064265 |
| 382 | Skin neoplasm bleeding | 10060712 |
| 383 | Skin ulcer haemorrhage | 10050377 |
| 384 | Small intestinal haemorrhage | 10052535 |
| 385 | Small intestinal ulcer haemorrhage | 10061550 |
| 386 | Soft tissue haemorrhage | 10051297 |
| 387 | Spermatic cord haemorrhage | 10065742 |
| 388 | Spinal cord haematoma | 10076051 |
| 389 | Spinal cord haemorrhage | 10048992 |
| 390 | Spinal epidural haematoma | 10050162 |
| 391 | Spinal epidural haemorrhage | 10049236 |
| 392 | Spinal subarachnoid haemorrhage | 10073564 |
| 393 | Spinal subdural haematoma | 10050164 |
| 394 | Spinal subdural haemorrhage | 10073563 |
| 395 | Spleen contusion | 10073533 |
| 396 | Splenic artery perforation | 10075738 |
| 397 | Splenic haematoma | 10041646 |
| 398 | Splenic haemorrhage | 10041647 |
| 399 | Splenic varices haemorrhage | 10068662 |
| 400 | Splinter haemorrhages | 10041663 |
| 401 | Spontaneous haematoma | 10065304 |
| 402 | Spontaneous haemorrhage | 10074557 |
| 403 | Stoma site haemorrhage | 10074508 |
| 404 | Stomatitis haemorrhagic | 10042132 |
| 405 | Subarachnoid haematoma | 10076701 |
| 406 | Subarachnoid haemorrhage | 10042316 |
| 407 | Subarachnoid haemorrhage neonatal | 10042317 |
| 408 | Subcapsular hepatic haematoma | 10083383 |
| 409 | Subcapsular renal haematoma | 10083385 |
| 410 | Subcapsular splenic haematoma | 10083384 |
| 411 | Subchorionic haematoma | 10072596 |
| 412 | Subchorionic haemorrhage | 10071010 |
| 413 | Subclavian artery perforation | 10075740 |
| 414 | Subclavian vein perforation | 10075743 |
| 415 | Subcutaneous haematoma | 10042345 |
| 416 | Subdural haematoma | 10042361 |
| 417 | Subdural haematoma evacuation | 10042363 |
| 418 | Subdural haemorrhage | 10042364 |
| 419 | Subdural haemorrhage neonatal | 10042365 |
| 420 | Subendocardial haemorrhage | 10082459 |
| 421 | Subgaleal haematoma | 10069510 |
| 422 | Subgaleal haemorrhage | 10080900 |
| 423 | Subretinal haematoma | 10071935 |
| 424 | Superior vena cava perforation | 10075741 |
| 425 | Testicular haemorrhage | 10051877 |
| 426 | Thalamus haemorrhage | 10058939 |
| 427 | Third stage postpartum haemorrhage | 10043449 |
| 428 | Thoracic haemorrhage | 10062744 |
| 429 | Thrombocytopenic purpura | 10043561 |
| 430 | Thrombotic thrombocytopenic purpura | 10043648 |
| 431 | Thyroid haemorrhage | 10064224 |
| 432 | Tongue haematoma | 10043959 |
| 433 | Tongue haemorrhage | 10049870 |
| 434 | Tonsillar haemorrhage | 10057450 |
| 435 | Tooth pulp haemorrhage | 10072228 |
| 436 | Tooth socket haemorrhage | 10064946 |
| 437 | Tracheal haemorrhage | 10062543 |
| 438 | Traumatic haematoma | 10044522 |
| 439 | Traumatic haemorrhage | 10053476 |
| 440 | Traumatic haemothorax | 10074487 |
| 441 | Traumatic intracranial haematoma | 10079013 |
| 442 | Traumatic intracranial haemorrhage | 10061387 |
| 443 | Tumour haemorrhage | 10049750 |
| 444 | Ulcer haemorrhage | 10061577 |
| 445 | Umbilical cord haemorrhage | 10064534 |
| 446 | Umbilical haematoma | 10068712 |
| 447 | Umbilical haemorrhage | 10045455 |
| 448 | Upper gastrointestinal haemorrhage | 10046274 |
| 449 | Ureteric haemorrhage | 10065743 |
| 450 | Urethral haemorrhage | 10049710 |
| 451 | Urinary bladder haematoma | 10083358 |
| 452 | Urinary bladder haemorrhage | 10046528 |
| 453 | Urogenital haemorrhage | 10050058 |
| 454 | Uterine haematoma | 10063875 |
| 455 | Uterine haemorrhage | 10046788 |
| 456 | Vaccination site bruising | 10069484 |
| 457 | Vaccination site haematoma | 10069472 |
| 458 | Vaccination site haemorrhage | 10069475 |
| 459 | Vaginal haematoma | 10046909 |
| 460 | Vaginal haemorrhage | 10046910 |
| 461 | Varicose vein ruptured | 10046999 |
| 462 | Vascular access site bruising | 10077767 |
| 463 | Vascular access site haematoma | 10077647 |
| 464 | Vascular access site haemorrhage | 10077643 |
| 465 | Vascular access site rupture | 10077652 |
| 466 | Vascular anastomotic haemorrhage | 10084092 |
| 467 | Vascular graft haemorrhage | 10077721 |
| 468 | Vascular pseudoaneurysm ruptured | 10053949 |
| 469 | Vascular purpura | 10047097 |
| 470 | Vascular rupture | 10053649 |
| 471 | Vein rupture | 10077110 |
| 472 | Venous haemorrhage | 10065441 |
| 473 | Venous perforation | 10075733 |
| 474 | Ventricle rupture | 10047279 |
| 475 | Vertebral artery perforation | 10075735 |
| 476 | Vessel puncture site bruise | 10063881 |
| 477 | Vessel puncture site haematoma | 10065902 |
| 478 | Vessel puncture site haemorrhage | 10054092 |
| 479 | Vitreous haematoma | 10071936 |
| 480 | Vitreous haemorrhage | 10047655 |
| 481 | Vulval haematoma | 10047756 |
| 482 | Vulval haematoma evacuation | 10047757 |
| 483 | Vulval haemorrhage | 10063816 |
| 484 | Withdrawal bleed | 10047998 |
| 485 | Wound haematoma | 10071504 |
| 486 | Wound haemorrhage | 10051373 |
