# Supplementary material for: Serious adverse reaction associated with the COVID-19 vaccines of BNT162b2, Ad26.COV2.S, and mRNA-1273: Gaining insight through the VAERS
Source: Front Pharmacol. 2022 Nov 7;13:921760. doi: 10.3389/fphar.2022.921760 (PMC9676979; doi:10.3389/fphar.2022.921760)
Supplement: Supplementary file 15 [file Table13.DOCX]

Supplementary Table 12 Serious adverse events and main complications following immunization associated with the COVID-19 vaccines of Ad26.COV2.S (Janssen).

| Died after vaccination (345 cases) | Cases | Percents |
| --- | --- | --- |
| Death | 262 | 75.94% |
| Dyspnoea | 37 | 10.72% |
| Unresponsive to stimuli | 31 | 8.99% |
| Cardiac arrest | 30 | 8.70% |
| Headache | 27 | 7.83% |
| Thrombosis | 27 | 7.83% |
| Malaise | 26 | 7.54% |
| Endotracheal intubation | 26 | 7.54% |
| Covid-19 | 23 | 6.67% |
| Sars-Cov-2 test positive | 23 | 6.67% |
| Threatens life (627 cases) | Cases | Percents |
| Pulmonary embolism | 128 | 20.41% |
| Dyspnoea | 120 | 19.14% |
| Headache | 99 | 15.79% |
| Blood test | 81 | 12.92% |
| Chest pain | 74 | 11.80% |
| Thrombosis | 71 | 11.32% |
| Computerised tomogram | 67 | 10.69% |
| Dizziness | 64 | 10.21% |
| Cerebrovascular accident | 63 | 10.05% |
| Deep vein thrombosis | 62 | 9.89% |
| Emergency visit (4,494 cases) | Cases | Percents |
| Headache | 1,044 | 23.23% |
| Dizziness | 878 | 19.54% |
| Dyspnoea | 713 | 15.87% |
| Nausea | 628 | 13.97% |
| Pyrexia | 623 | 13.86% |
| Fatigue | 588 | 13.08% |
| Pain in extremity | 569 | 12.66% |
| Pain | 565 | 12.57% |
| Chills | 510 | 11.35% |
| Blood test | 427 | 9.50% |
| Hospitalization (1,777 cases) | Cases | Percents |
| Headache | 307 | 17.28% |
| Dyspnoea | 267 | 15.03% |
| Pulmonary embolism | 224 | 12.61% |
| Pyrexia | 195 | 10.97% |
| Computerised tomogram | 171 | 9.62% |
| Nausea | 158 | 8.89% |
| Thrombosis | 157 | 8.84% |
| Blood test | 154 | 8.67% |
| Cerebrovascular accident | 154 | 8.67% |
| Dizziness | 151 | 8.50% |

For people received Ad26.COV2.S, 345 people died after the vaccine (2.06 per 1,000, 345/167,457), 627 people (3.74 per 1,000, 627/167,457) suffered from life-threatening AEFI, and people visited emergency rooms and hospitalization were 4,494 (26.84 per 1,000, 4,494/167,457) and 1,777 (10.61 per 1,000, 1,777/167,457), respectively.
