# Supplementary material for: Serious adverse reaction associated with the COVID-19 vaccines of BNT162b2, Ad26.COV2.S, and mRNA-1273: Gaining insight through the VAERS
Source: Front Pharmacol. 2022 Nov 7;13:921760. doi: 10.3389/fphar.2022.921760 (PMC9676979; doi:10.3389/fphar.2022.921760)
Supplement: Supplementary file 16 [file Table4.DOCX]

Supplementary Table 3 The preferred term of thrombocytopenia used in this study.

|  | **Preferred term** | **Code** |
| --- | --- | --- |
| 1 | Acquired amegakaryocytic thrombocytopenia | 10076747 |
| 2 | Heparin-induced thrombocytopenia | 10062506 |
| 3 | Megakaryocytes decreased | 10027119 |
| 4 | Platelet count decreased | 10035528 |
| 5 | Platelet maturation arrest | 10035537 |
| 6 | Platelet production decreased | 10035540 |
| 7 | Platelet toxicity | 10059440 |
| 8 | Thrombocytopenia | 10043554 |
| 9 | Megakaryocytes abnormal | 10027118 |
| 10 | Platelet count abnormal | 10035526 |
| 11 | Platelet disorder | 10035532 |
| 12 | Plateletcrit abnormal | 10064785 |
| 13 | Plateletcrit decreased | 10064784 |
| 14 | Thrombocytopenia neonatal | 10043557 |
