# Supplementary material for: Serious adverse reaction associated with the COVID-19 vaccines of BNT162b2, Ad26.COV2.S, and mRNA-1273: Gaining insight through the VAERS
Source: Front Pharmacol. 2022 Nov 7;13:921760. doi: 10.3389/fphar.2022.921760 (PMC9676979; doi:10.3389/fphar.2022.921760)
Supplement: Supplementary file 17 [file Table11.DOCX]

Supplementary Table 10 The preferred term of convulsions used in this study.

| **Convulsions events** | **Preferred term** | **Code** |
| --- | --- | --- |
| 1 | 1p36 deletion syndrome | 10082398 |
| 2 | 2-Hydroxyglutaric aciduria | 10078971 |
| 3 | Acquired epileptic aphasia | 10052075 |
| 4 | Acute encephalitis with refractory, repetitive partial seizures | 10076948 |
| 5 | Alcoholic seizure | 10056347 |
| 6 | Alpers disease | 10083857 |
| 7 | Aspartate-glutamate-transporter deficiency | 10079140 |
| 8 | Atonic seizures | 10003628 |
| 9 | Atypical benign partial epilepsy | 10056699 |
| 10 | Automatism epileptic | 10003831 |
| 11 | Autonomic seizure | 10049612 |
| 12 | Baltic myoclonic epilepsy | 10054895 |
| 13 | Benign familial neonatal convulsions | 10067866 |
| 14 | Benign rolandic epilepsy | 10070530 |
| 15 | Biotinidase deficiency | 10071434 |
| 16 | CDKL5 deficiency disorder | 10083005 |
| 17 | CEC syndrome | 10083749 |
| 18 | Change in seizure presentation | 10075606 |
| 19 | Clonic convulsion | 10053398 |
| 20 | Congenital bilateral perisylvian syndrome | 10082716 |
| 21 | Convulsion in childhood | 10052391 |
| 22 | Convulsions local | 10010920 |
| 23 | Convulsive threshold lowered | 10010927 |
| 24 | CSWS syndrome | 10078827 |
| 25 | Deja vu | 10012177 |
| 26 | Double cortex syndrome | 10073490 |
| 27 | Dreamy state | 10013634 |
| 28 | Drug withdrawal convulsions | 10013752 |
| 29 | Early infantile epileptic encephalopathy with burst-suppression | 10071545 |
| 30 | Eclampsia | 10014129 |
| 31 | Epilepsy | 10015037 |
| 32 | Epilepsy surgery | 10079824 |
| 33 | Epilepsy with myoclonic-atonic seizures | 10081179 |
| 34 | Epileptic aura | 10015049 |
| 35 | Epileptic psychosis | 10059232 |
| 36 | Febrile convulsion | 10016284 |
| 37 | Febrile infection-related epilepsy syndrome | 10079438 |
| 38 | Focal dyscognitive seizures | 10079424 |
| 39 | Frontal lobe epilepsy | 10049424 |
| 40 | Gelastic seizure | 10082918 |
| 41 | Generalised onset non-motor seizure | 10083376 |
| 42 | Generalised tonic-clonic seizure | 10018100 |
| 43 | Glucose transporter type 1 deficiency syndrome | 10078727 |
| 44 | GM2 gangliosidosis | 10083933 |
| 45 | Grey matter heterotopia | 10082084 |
| 46 | Hemimegalencephaly | 10078100 |
| 47 | Hyperglycaemic seizure | 10071394 |
| 48 | Hypocalcaemic seizure | 10072456 |
| 49 | Hypoglycaemic seizure | 10048803 |
| 50 | Hyponatraemic seizure | 10073183 |
| 51 | Idiopathic generalised epilepsy | 10071081 |
| 52 | Infantile spasms | 10021750 |
| 53 | Juvenile myoclonic epilepsy | 10071082 |
| 54 | Lafora's myoclonic epilepsy | 10054030 |
| 55 | Lennox-Gastaut syndrome | 10048816 |
| 56 | Migraine-triggered seizure | 10076676 |
| 57 | Molybdenum cofactor deficiency | 10069687 |
| 58 | Multiple subpial transection | 10079825 |
| 59 | Myoclonic epilepsy | 10054859 |
| 60 | Myoclonic epilepsy and ragged-red fibres | 10069825 |
| 61 | Neonatal epileptic seizure | 10082068 |
| 62 | Neonatal seizure | 10082067 |
| 63 | Partial seizures | 10061334 |
| 64 | Partial seizures with secondary generalisation | 10056209 |
| 65 | Petit mal epilepsy | 10034759 |
| 66 | Polymicrogyria | 10073489 |
| 67 | Post stroke epilepsy | 10076982 |
| 68 | Post stroke seizure | 10076981 |
| 69 | Postictal headache | 10052470 |
| 70 | Postictal paralysis | 10052469 |
| 71 | Postictal psychosis | 10070669 |
| 72 | Postictal state | 10048727 |
| 73 | Post-traumatic epilepsy | 10036312 |
| 74 | Schizencephaly | 10073487 |
| 75 | Seizure | 10039906 |
| 76 | Seizure anoxic | 10039907 |
| 77 | Seizure cluster | 10071350 |
| 78 | Seizure like phenomena | 10071048 |
| 79 | Severe myoclonic epilepsy of infancy | 10073677 |
| 80 | Simple partial seizures | 10040703 |
| 81 | Status epilepticus | 10041962 |
| 82 | Sudden unexplained death in epilepsy | 10063894 |
| 83 | Temporal lobe epilepsy | 10043209 |
| 84 | Tonic clonic movements | 10051171 |
| 85 | Tonic convulsion | 10043994 |
| 86 | Tonic posturing | 10075125 |
| 87 | Topectomy | 10073488 |
| 88 | Transient epileptic amnesia | 10081728 |
| 89 | Tuberous sclerosis complex | 10080584 |
| 90 | Uncinate fits | 10045476 |
| 91 | Amygdalohippocampectomy | 10071707 |
| 92 | Aura | 10003791 |
| 93 | Corpus callosotomy | 10073491 |
| 94 | Drop attacks | 10013643 |
| 95 | Foaming at mouth | 10062654 |
| 96 | Focal cortical resection | 10083272 |
| 97 | Narcolepsy | 10028713 |
| 98 | Preictal state | 10073854 |
| 99 | Seizure prophylaxis | 10081601 |
| 100 | Tongue biting | 10050467 |
