# Supplementary material for: Serious adverse reaction associated with the COVID-19 vaccines of BNT162b2, Ad26.COV2.S, and mRNA-1273: Gaining insight through the VAERS
Source: Front Pharmacol. 2022 Nov 7;13:921760. doi: 10.3389/fphar.2022.921760 (PMC9676979; doi:10.3389/fphar.2022.921760)
Supplement: Supplementary file 18 [file Table6.DOCX]

Supplementary Table 5 The preferred term of cardiac failure used in this study.

|  | **Preferred term** | **Code** |
| --- | --- | --- |
| 1 | Acute left ventricular failure | 10063081 |
| 2 | Acute pulmonary oedema | 10001029 |
| 3 | Acute right ventricular failure | 10063082 |
| 4 | Cardiac asthma | 10007522 |
| 5 | Cardiac failure | 10007554 |
| 6 | Cardiac failure acute | 10007556 |
| 7 | Cardiac failure chronic | 10007558 |
| 8 | Cardiac failure congestive | 10007559 |
| 9 | Cardiac failure high output | 10007560 |
| 10 | Cardiogenic shock | 10007625 |
| 11 | Cardiohepatic syndrome | 10082480 |
| 12 | Cardiopulmonary failure | 10051093 |
| 13 | Cardiorenal syndrome | 10068230 |
| 14 | Chronic left ventricular failure | 10063083 |
| 15 | Chronic right ventricular failure | 10063084 |
| 16 | Congestive hepatopathy | 10084058 |
| 17 | Cor pulmonale | 10010968 |
| 18 | Cor pulmonale acute | 10010969 |
| 19 | Cor pulmonale chronic | 10010970 |
| 20 | Ejection fraction decreased | 10050528 |
| 21 | Hepatojugular reflux | 10051448 |
| 22 | Left ventricular failure | 10024119 |
| 23 | Low cardiac output syndrome | 10024899 |
| 24 | Neonatal cardiac failure | 10049780 |
| 25 | Obstructive shock | 10073708 |
| 26 | Pulmonary oedema | 10037423 |
| 27 | Pulmonary oedema neonatal | 10050459 |
| 28 | Radiation associated cardiac failure | 10076203 |
| 29 | Right ventricular ejection fraction decreased | 10075337 |
| 30 | Right ventricular failure | 10039163 |
| 31 | Ventricular failure | 10060953 |
| 32 | Artificial heart implant | 10072066 |
| 33 | Atrial natriuretic peptide abnormal | 10053410 |
| 34 | Atrial natriuretic peptide increased | 10053412 |
| 35 | Bendopnoea | 10077819 |
| 36 | Brain natriuretic peptide abnormal | 10053408 |
| 37 | Brain natriuretic peptide increased | 10053405 |
| 38 | Cardiac cirrhosis | 10054936 |
| 39 | Cardiac contractility modulation therapy | 10077454 |
| 40 | Cardiac device reprogramming | 10081886 |
| 41 | Cardiac dysfunction | 10079751 |
| 42 | Cardiac index decreased | 10007577 |
| 43 | Cardiac output decreased | 10007595 |
| 44 | Cardiac resynchronisation therapy | 10059862 |
| 45 | Cardiac ventriculogram abnormal | 10053447 |
| 46 | Cardiac ventriculogram left abnormal | 10053499 |
| 47 | Cardiac ventriculogram right abnormal | 10053444 |
| 48 | Cardiomegaly | 10007632 |
| 49 | Cardio-respiratory distress | 10049874 |
| 50 | Cardiothoracic ratio increased | 10007646 |
| 51 | Central venous pressure increased | 10007980 |
| 52 | Coronary sinus dilatation | 10082615 |
| 53 | Diastolic dysfunction | 10052337 |
| 54 | Dilatation ventricular | 10013012 |
| 55 | Dyspnoea paroxysmal nocturnal | 10013974 |
| 56 | Heart transplant | 10019314 |
| 57 | Hepatic vein dilatation | 10069112 |
| 58 | Implantable cardiac monitor replacement | 10082009 |
| 59 | Intracardiac pressure increased | 10079904 |
| 60 | Jugular vein distension | 10059865 |
| 61 | Left ventricular diastolic collapse | 10080987 |
| 62 | Left ventricular dilatation | 10050043 |
| 63 | Left ventricular dysfunction | 10049694 |
| 64 | Left ventricular enlargement | 10050581 |
| 65 | Lower respiratory tract congestion | 10075565 |
| 66 | Myocardial depression | 10069140 |
| 67 | Nocturnal dyspnoea | 10049235 |
| 68 | N-terminal prohormone brain natriuretic peptide abnormal | 10071660 |
| 69 | N-terminal prohormone brain natriuretic peptide increased | 10071662 |
| 70 | Oedema | 10030095 |
| 71 | Oedema blister | 10080039 |
| 72 | Oedema due to cardiac disease | 10049632 |
| 73 | Oedema neonatal | 10061317 |
| 74 | Oedema peripheral | 10030124 |
| 75 | Orthopnoea | 10031123 |
| 76 | Peripheral oedema neonatal | 10049779 |
| 77 | Peripheral swelling | 10048959 |
| 78 | Post cardiac arrest syndrome | 10078202 |
| 79 | Prohormone brain natriuretic peptide abnormal | 10077783 |
| 80 | Prohormone brain natriuretic peptide increased | 10077781 |
| 81 | Pulmonary congestion | 10037368 |
| 82 | Right ventricular diastolic collapse | 10079613 |
| 83 | Right ventricular dilatation | 10074222 |
| 84 | Right ventricular dysfunction | 10058597 |
| 85 | Right ventricular enlargement | 10050582 |
| 86 | Scan myocardial perfusion abnormal | 10061501 |
| 87 | Stroke volume decreased | 10042246 |
| 88 | Surgical ventricular restoration | 10078218 |
| 89 | Systolic dysfunction | 10071436 |
| 90 | Venous pressure increased | 10047236 |
| 91 | Venous pressure jugular abnormal | 10047238 |
| 92 | Venous pressure jugular increased | 10047240 |
| 93 | Ventricular assist device insertion | 10052371 |
| 94 | Ventricular compliance decreased | 10080992 |
| 95 | Ventricular dysfunction | 10059056 |
| 96 | Ventricular dyssynchrony | 10071186 |
| 97 | Wall motion score index abnormal | 10079016 |
