# Supplementary material for: Serious adverse reaction associated with the COVID-19 vaccines of BNT162b2, Ad26.COV2.S, and mRNA-1273: Gaining insight through the VAERS
Source: Front Pharmacol. 2022 Nov 7;13:921760. doi: 10.3389/fphar.2022.921760 (PMC9676979; doi:10.3389/fphar.2022.921760)
Supplement: Supplementary file 19 [file Table14.DOCX]

Supplementary Table 13 Serious adverse events and main complications following immunization associated with COVID-19 vaccines of mRNA-1273 (Moderna).

| Died after vaccination (2,077 cases) | Cases | Percents |
| --- | --- | --- |
| Death | 1,746 | 84.06% |
| Dyspnoea | 216 | 10.40% |
| Unresponsive to stimuli | 172 | 8.28% |
| Cardiac arrest | 155 | 7.46% |
| Resuscitation | 139 | 6.69% |
| Fatigue | 121 | 5.83% |
| Pyrexia | 120 | 5.78% |
| Covid-19 | 116 | 5.58% |
| Vomiting | 104 | 5.01% |
| Asthenia | 103 | 4.96% |
| Threatens life (1,712 cases) | Cases | Percents |
| Dyspnoea | 352 | 20.56% |
| Pulmonary embolism | 215 | 12.56% |
| Pyrexia | 191 | 11.16% |
| Blood test | 167 | 9.75% |
| Headache | 162 | 9.46% |
| Cerebrovascular accident | 147 | 8.59% |
| Computerised tomogram | 144 | 8.41% |
| Dizziness | 141 | 8.24% |
| Fatigue | 140 | 8.18% |
| Nausea | 138 | 8.06% |
| Emergency visit (13,092 case) | Cases | Percents |
| Dizziness | 2,247 | 17.16% |
| Dyspnoea | 1,956 | 14.94% |
| Headache | 1,647 | 12.58% |
| Nausea | 1,611 | 12.31% |
| Pyrexia | 1,355 | 10.35% |
| Fatigue | 1,203 | 9.19% |
| Pain | 1,183 | 9.04% |
| Chills | 1,105 | 8.44% |
| Electrocardiogram | 968 | 7.39% |
| Rash | 850 | 6.49% |
| Hospitalization (5,979 case) | Cases | Percents |
| Dyspnoea | 890 | 14.89% |
| Pyrexia | 786 | 13.15% |
| Asthenia | 566 | 9.47% |
| Headache | 533 | 8.91% |
| Fatigue | 520 | 8.70% |
| Nausea | 509 | 8.51% |
| Chills | 462 | 7.73% |
| Dizziness | 453 | 7.58% |
| Vomiting | 452 | 7.56% |
| Blood test | 414 | 6.92% |

For people who got the mRNA-1273 vaccination, 2,077 people (3.88 per 1,000, 2,077/535,126) died after the vaccine including 116 (5.58%) died of the COVID-19, 1,712 people (3.20 per thousand, 1,712/535,126) suffered from life-threatening AEFI and 13,092 (24.46 per 1,000, 13,092/535,126) and 5,979 (11.17 per 1,000, 5,979/535,126) people visited emergency rooms and hospitalization.
