# Supplementary material for: Serious adverse reaction associated with the COVID-19 vaccines of BNT162b2, Ad26.COV2.S, and mRNA-1273: Gaining insight through the VAERS
Source: Front Pharmacol. 2022 Nov 7;13:921760. doi: 10.3389/fphar.2022.921760 (PMC9676979; doi:10.3389/fphar.2022.921760)
Supplement: Supplementary file 20 [file Table10.DOCX]

Supplementary Table 9 The preferred term of acute pancreatitis used in this study.

|  | **Preferred term** | **Code** |
| --- | --- | --- |
| 1 | Cullen's sign | 10059029 |
| 2 | Grey Turner's sign | 10075426 |
| 3 | Haemorrhagic necrotic pancreatitis | 10076058 |
| 4 | Hereditary pancreatitis | 10056976 |
| 5 | Immune-mediated pancreatitis | 10083072 |
| 6 | Ischaemic pancreatitis | 10066127 |
| 7 | Oedematous pancreatitis | 10052400 |
| 8 | Pancreatic abscess | 10048984 |
| 9 | Pancreatic cyst drainage | 10082531 |
| 10 | Pancreatic haemorrhage | 10033625 |
| 11 | Pancreatic necrosis | 10058096 |
| 12 | Pancreatic phlegmon | 10056975 |
| 13 | Pancreatic pseudoaneurysm | 10081762 |
| 14 | Pancreatic pseudocyst | 10033635 |
| 15 | Pancreatic pseudocyst drainage | 10033636 |
| 16 | Pancreatic pseudocyst haemorrhage | 10083813 |
| 17 | Pancreatic pseudocyst rupture | 10083811 |
| 18 | Pancreatitis | 10033645 |
| 19 | Pancreatitis acute | 10033647 |
| 20 | Pancreatitis haemorrhagic | 10033650 |
| 21 | Pancreatitis necrotising | 10033654 |
| 22 | Pancreatitis relapsing | 10033657 |
| 23 | Pancreatorenal syndrome | 10056277 |
